# ARTP/EMS-combined multiple mutagenesis efficiently improved production of raw starch-degrading enzymes in *Penicillium oxalicum* and characterization of the enzyme-hyperproducing mutant

**DOI:** 10.1186/s13068-020-01826-5

**Published:** 2020-11-11

**Authors:** Li-Sha Gu, Ming-Zhu Tan, Shi-Huan Li, Ting Zhang, Qi-Qiang Zhang, Cheng-Xi Li, Xue-Mei Luo, Jia-Xun Feng, Shuai Zhao

**Affiliations:** State Key Laboratory for Conservation and Utilization of Subtropical Agro-Bioresources, Guangxi Research Center for Microbial and Enzyme Engineering Technology, College of Life Science and Technology, Guangxi University, 100 Daxue Road, Nanning, 530004 Guangxi People’s Republic of China

**Keywords:** Raw starch-degrading enzymes, ARTP/EMS-combined mutagenesis, *Penicillium oxalicum*

## Abstract

**Background:**

Application of raw starch-degrading enzymes (RSDEs) in starch processing for biofuel production can effectively reduce energy consumption and processing costs. RSDEs are generally produced by filamentous fungi, such as *Penicillium oxalicum*, but with very low yields, which seriously hampers industrialization of raw starch processing. Breeding assisted by random mutagenesis is an efficient way to improve fungal enzyme production.

**Results:**

A total of 3532 *P. oxalicum* colonies were generated after multiple rounds of mutagenesis, by atmospheric and room-temperature plasma (ARTP) and/or ethyl methanesulfonate (EMS). Of these, one mutant A2-13 had the highest RSDE activity of 162.7 U/mL, using raw cassava flour as substrate, a yield increase of 61.1%, compared with that of the starting strain, OX*PoxGA15A*. RSDE activity of A2-13 further increased to 191.0 U/mL, through optimization of culture conditions. Increased expression of major amylase genes, including the raw starch-degrading glucoamylase gene, *PoxGA15A,* and its regulatory gene, *PoxAmyR*, as well as several single-nucleotide polymorphisms in the A2-13 genome, were detected by real-time reverse transcription quantitative PCR and genomic re-sequencing, respectively. In addition, crude RSDEs produced by A2-13, combined with commercial α-amylase, could efficiently digest raw corn flour and cassava flour at 40 °C.

**Conclusions:**

Overall, ARTP/EMS-combined mutagenesis effectively improved fungal RSDE yield. An RSDE-hyperproducing mutant, A2-13, was obtained, and its RSDEs could efficiently hydrolyze raw starch, in combination with commercial α-amylase at low temperature, which provides a useful RSDE resource for future starch processing.

## Background

Plant biomass biorefineries use renewable and relatively inexpensive raw materials as feedstocks for processing into value-added biofuels and chemicals, with potential benefits for industry and the environment. Biorefinery processes can help alleviate problems associated with industrial chemicals and fossil fuels, such as increasing energy costs, environmental pollution and climate change [1, 2]. A major challenge, however, for biorefineries, is the high cost of enzymes used for biomass degradation into fermentable sugars, which results in poor profitability [1].

Bioethanol production by cassava starch biorefineries has attracted considerable interest because of the competitive advantages of cassava, such as abundant availability, low cost, and non-competition with direct food/feed supplies [3]. Conventional enzymatic starch processing requires an initial high-temperature liquefaction step, using thermostable α-amylase, followed by saccharification with glucoamylase, after cooling below the starch gelatinization temperature, then fermentation to produce bioethanol [4]. Even these enzymatic processes require a large energy input and special equipment, which increases processing costs. The liquefaction step accounts for approximately 30‒40% of the total cost of bioethanol production [5].

Amylase for starch processing accounts for the majority of the industrial enzyme market and consists of four related enzymes, α-amylase (EC 3.2.1.1), glucoamylase (EC 3.2.1.3), α-glucosidase (EC 3.2.1.20), and 1,4-a-glucanbranching enzyme (EC 2.4.1.18) (https://www.cazy.org/). α-Amylase and glucoamylase are commonly used in combination for starch processing. α-amylase breaks α-1,4-glycosidic bonds into amylopectin, or amylose straight chains, to release straight-chain and branched oligosaccharides of various lengths. Glucoamylase can cleave both α-1,4- or α-1,6-glucosidic bonds at the non-reducing ends of starch chains, or dextrins, to release glucose [6, 7].

Remarkably, a few amylase proteins contain starch-binding domains (SBDs) that allow them to bind to the surface of raw starch granules [8], thereby efficiently and directly digesting granular, raw starch into glucose, even below the gelatinization temperature of starch. Those amylases are known as raw starch-degrading enzymes (RSDEs) and have potential applications in starch processing. In the natural environment, RSDEs are mainly secreted by filamentous fungi, but with low yields, which have not yet met the quantitative and cost requirements for large-scale industrialization of raw starch biorefining.

Classical physical or chemical mutagenesis is an efficient strategy for breeding of fungal strains, in particular, for improvement of enzyme yields. The main mutagens used are ethyl methanesulfonate (EMS), nitroglycerin, UV-radiation, and “atmospheric and room temperature plasma” (ARTP) [9–12]. The chemical agent EMS alkylates nucleotides at random positions, thereby resulting in transition mutations [13]. ARTP can change the structure and permeability of the cell wall and plasma membrane, by generation of plasma jets, which causes DNA damage; ARTP is a recently developed and effective mutation technique [14]. The use of EMS combined with UV treatment has been reported [9, 10], whereas EMS combined with ARTP has not, to the best of our knowledge.

In this study, we employed multiple rounds of ARTP/EMS-combined mutagenesis to improve RSDE production in *P. oxalicum*. The starting strain used was the engineered strain OX*PoxGA15A,* derived from the parental strain ∆*PoxKu70* via over-expressing a raw-starch-degrading glucoamylase gene *PoxGA15A* [11, 15, 16]. Subsequently, culture conditions were optimized for the resulting mutant with the highest RSDE production and enzymatic hydrolysis efficiency against raw starch flour. Both genome re-sequencing and real-time reverse transcription quantitative PCR (RT-qPCR) were employed to analyze single-nucleotide polymorphisms and transcriptional levels of genes encoding major RSDEs in the isolated mutants.

## Results and discussion

### Development of a two-layer agar gel diffusion method for rapidly screening for RSDE-hyperproducing mutants

**Fig. 1:**
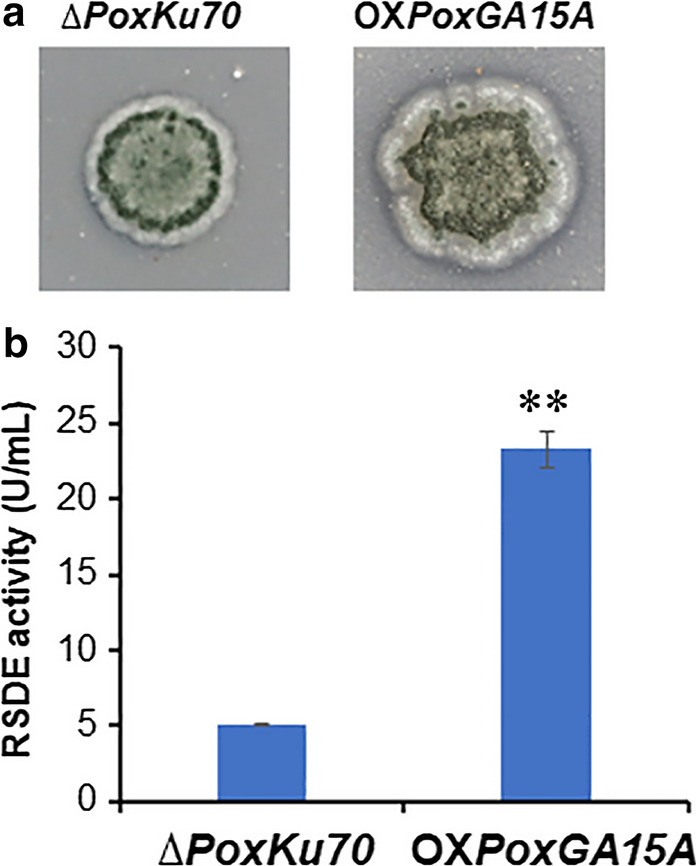
Phenotypic investigation of *P. oxalicum* strains ∆*PoxKu70* and OX*PoxGA15A,* grown on a two-layer agar gel plate (**a**) and their RSDE activity in liquid medium (**b**). The two-layer agar gel plate consisted of an upper layer containing ball-milled Avicel (0.5% w/v) and natural raw cassava flour (RNCF, 1% w/v), and a lower layer of MMM without carbon source. Carbon sources used in liquid medium were the same as that in the top layer of the two-layer plate. RSDE activity was determined using RNCF as the substrate. ** (*p* ≤ 0.01) indicates significant difference between the OX*PoxGA15A* and ∆*PoxKu70* by Student’s *t* test. *RSDE* raw starch-degrading enzyme, *MMM* modified minimal medium

To optimize screening efficiency for mutants with improved RSDE production, a high-throughput screening method using two-layer agar gels was developed. A two-layer agar gel was prepared, containing raw natural cassava flour (RNCF) and ball-milled Avicel, at a series of different ratios, as the top layer, and modified minimal medium (MMM), without carbon source as the bottom layer. RNCF flour was ground directly from freshly harvested cassava tubers after drying, without any other pretreatments, such as cellulose removal. RNCF is very similar to the cassava flour used in the starch industry and different from edible raw starch available from farmer’s markets. As expected, Avicel stimulated the expression of cellulase and xylanase genes, as well as induction of the pPoxEgCel5B promoter, which controls over-expression of the RSDG gene, *PoxGA15A* in OX*PoxGA15A* [15], whereas RNCF induced the expression of amylase genes, including RSDE genes. The two-layer agar gel containing 0.5% Avicel and 1.0% RNCF exhibited clear zones around colonies of OX*PoxGA15A* and the parental strain ∆*PoxKu70*, the clear zones around the OX*PoxGA15A* colonies being slightly bigger (Fig. 1a). When inoculated into liquid MMM containing 0.5% Avicel and 1.0% RNCF, the RSDE activity of OX*PoxGA15A* was 362.6% higher than that of ∆*PoxKu70* (*p* < 0.01; Fig. 1b). These data confirmed that the two-layer agar gel was an effective way to screen for RSDE hyperproducers, after random mutagenesis.

### ARTP/EMS-mediated mutagenesis and screening for RSDE hyperproducers

ARTP/EMS-mediated mutagenesis was performed in three steps, i.e., (1) three rounds of EMS, (2) one round of ARTP, and (3) one round of ARTP combined with EMS (Additional file 1: Fig. S1). Prior to EMS and/or ARTP mutagenesis, the optimal lethality of EMS against the starting strain OX*PoxGA15A* was determined at 1, 2, 4, 8, and 12 h after treatment. Treatment with 2% EMS for 8 h resulted in 93.6% lethality (i.e., cell death), whereas treatment for 12 h resulted in 100% lethality (Additional file 2: Fig. S2A).

**Fig. 2:**
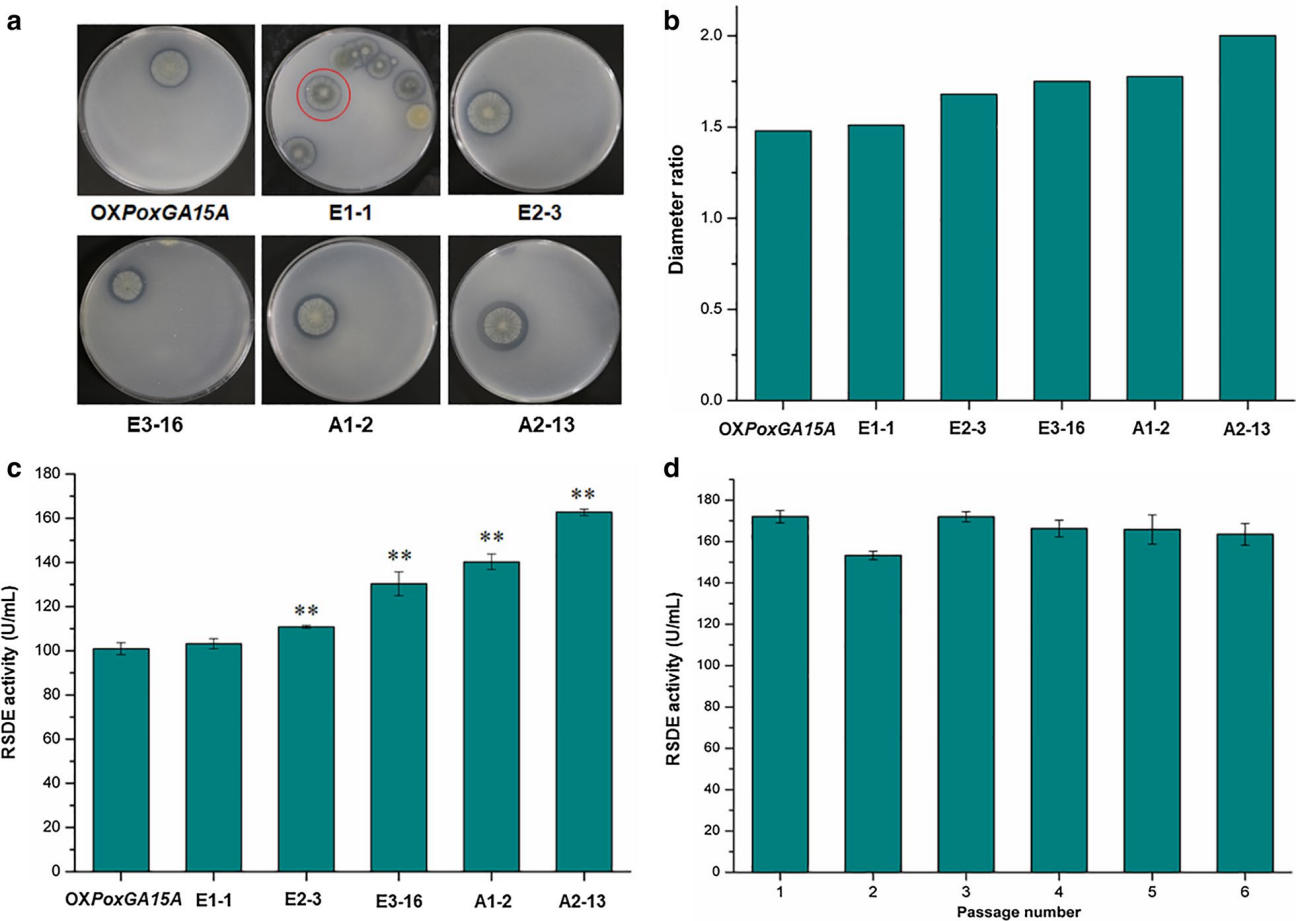
Investigation of potential *P. oxalicum* mutants isolated from each round of mutagenesis. **a** Clear zones on the two-layer agar gel plates after 8 days. Red circle indicates the isolated colony used for the next round of mutagenesis. **b** Diameter ratio between clear zone and colony. **c** RSDE activity. **d** RSDE activity of the strain A2-13 sub-cultured six times. RSDE activity was determined using RNCF as the substrate. ** (*p* ≤ 0.01) indicates significant difference between the isolates and the starting strain OX*PoxGA15A* by Student’s *t* test. *RSDE* raw starch-degrading enzyme, *RNCF* natural raw cassava flour

The lethality of ARTP against mutant E3-16 (see following description) was also determined, after 350, 400, 450, 500, and 550 s of treatment at a radio-frequency power input of 130 W and a flow rate of 10 L/min. The lethality increased with treatment time and treatment for 500 s resulted in 95.1% lethality (Additional file 2: Fig. S2B).

When cultured for 8 days after treatment, a total of 3532 colonies were observed on the two-layer agar plates, including 1616 colonies from three rounds of EMS treatment, 1120 from ARTP treatment and 796 from the ARTP/EMS combination (Additional file 1: Fig. S1). Comparison of the diameter ratio between each colony and its clear zone selected the mutants designated as E1-1, E2-3, E3-16, A1-2, and A2-13 as having the largest ratios after each round of mutagenesis; their diameter ratios ranged from 1.51 to 2.0, compared with 1.48 for the original strain, OX*PoxGA15A* (Fig. 2a, b). Measurement of enzymatic activities determined the RSDE activity of each mutant, using RNCF as the substrate, ranged from 103.2 to 162.7 U/mL, when cultured in liquid MMM containing 4% wheat bran plus 1% Avicel, which was consistent with its diameter ratio (Fig. 2c). A2-13 had the highest RSDE activity and the largest diameter ratio, so it was selected for further study.

RSDE production by A2-13 was 61.0% higher than that of the starting strain OX*PoxGA15A,* after multiple rounds of ARTP/EMS mutagenesis. This appears to be attributable to mutant selection using the two-layer agar screening plates, containing both cassava starch and Avicel. The clear zones on the screening plates indicated degradation of both starch and Avicel.

To evaluate the genetic stability of mutant A2-13, regarding enzyme production, the RSDE activities, using RNCF as the substrate, were measured after each of six successive sub-cultures. No significant change in RSDE production was observed after any of the sub-cultures (Fig. 2d).

### Optimization of liquid culture conditions

The secretion of plant biomass-degrading enzymes (i.e., amylase, cellulase, and xylanase) by filamentous fungi is closely related to the characteristics of the lignocellulosic substrates in media, such as physical properties and chemical composition, as well as culture-related factors, such as the culture set-up and substrate loading [1, 17]. Therefore, to further improve RSDE production by mutant A2-13, the following liquid culture parameters were optimized: the initial pH of the medium, incubation temperature, composition of carbon source and composition ratio, nitrogen source, and concentration of spore inoculum. The optimal conditions found were as follows: initial pH of medium, 5.5; incubation temperature, 28 °C; composition of carbon source, wheat bran plus Avicel (2:3 w/w); nitrogen source, 5 g/L NH_4_NO_3_; and concentration of spore inoculum, 1% (v/v) inoculum with 10^8^ spores/mL (Additional file 3: Fig. S3). When cultured under these optimal culture conditions, mutant A2-13 produced 191.0 U/mL of RSDEs, which was 17.4% and 89.1% higher than those of A2-13 and OX*PoxGA15A* under non-optimal culture conditions (*p* < 0.01), respectively. This was also far higher RSDE production than by *P. oxalicum* strain GXU20 (20 U/mL), using the same substrate [17]. The production of RSDE by A2-13 against RNCF, under the optimal conditions, is the highest reported to date. Other reports of RSDE activities using different raw starch substrates, such as processed cassava starch, potato starch, or uncooked soluble starch, are not directly comparable (Table 1).

**Table 1:** Comparison of amylase activity of *P. oxalicum* mutant A2-13 with other microbial strains in the literature

Microorganisms	Starch substrate	Enzyme activity (U/mL)	References
*P. oxalicum* A2-13	Nature raw cassava flour	191.0	This study
*P. oxalicum OXPoxGA15A*	Nature raw cassava flour	101.0	This study
*P. oxalicum OXPoxGA15A*	Processed raw cassava flour	241.6	[15]
*P. oxalicum* Δ*PoxKu70*	Processed raw cassava flour	55.1	[15]
*P. oxalicum* GXU20	Nature raw cassava flour	20.0	[17]
*Aspergillus fumigatus* CFU-01	Soluble starch	24.9	[18]
*Aspergillus *sp. MZA-3	Raw cassava starch	3.3	[19]
*Thermomucor indicae-seudaticae*	Uncooked soluble starch	34.0	[20]
*Rhizopus *sp. A-11	Uncooked soluble starch	190.0	[21]
*Aureobasidium pullulans* N13d	Raw potato starch	10.0	[22]

### Properties of crude RSDE produced by the mutant A2-13

**Fig. 3:**
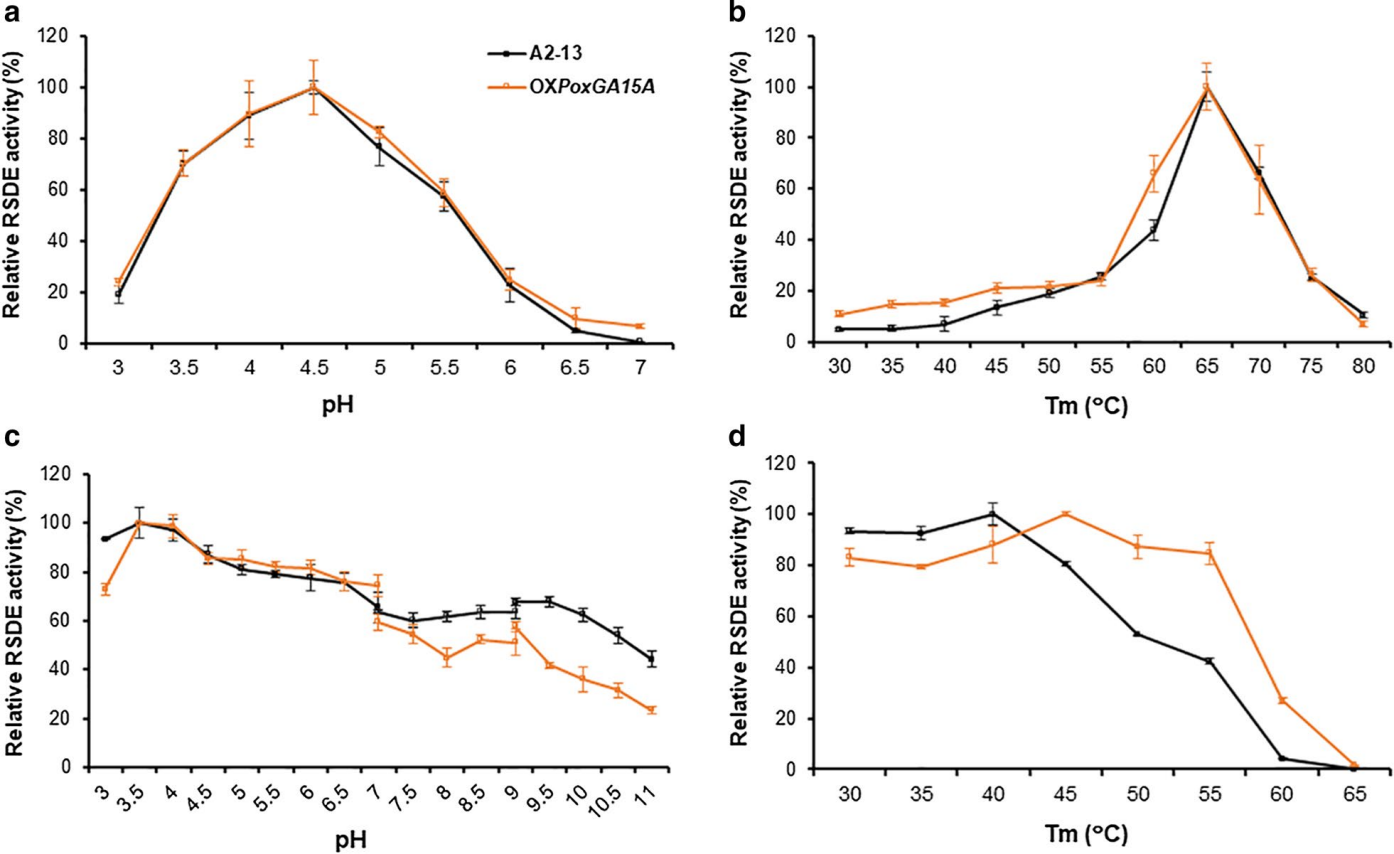
Effects of pH and temperature on RSDE activity of *P. oxalicum* strains A2-13 and OX*PoxGA15A*. **a** pH profile of RSDE activity. Enzyme activity was measured at pHs between 3.0 and 7.0 in 0.1 M citrate–phosphate buffer at 37 °C. **b** Temperature profile of RSDE activity. **c** Effect of pH on RSDE stability. **d** Thermal stability of RSDE activity. *P. oxalicum* strains were cultured for 6 days under the optimal culture conditions. In **a** and **b**, the highest RSDE activity of A2-13 and OX*PoxGA15A* was set as 100%. In **c** and **d**, the RSDE activity of untreated A2-13 and OX*PoxGA15A* was set as 100%. Each experiment was independently performed three times. Each data point represents mean ± SD

To investigate the effects of ARTP/EMS-combined mutagenesis on the properties of the crude enzymes produced, the optimal pH and temperature of the crude enzymes were measured in comparison with those of OX*PoxGA15A*. The optimum pH and temperature, with RNCF as substrate, were 4.5 and 65 °C, respectively, which were similar to those of crude enzymes from OX*PoxGA15A* (Fig. 3a, b). Moreover, pH and heat stability analyses revealed that crude RSDE from A2-13 was highly stable when degrading RNCF under acidic conditions, again similar to RSDE from OX*PoxGA15A*. Interestingly, crude RSDE from strain A2-13 also had improved tolerance to alkaline conditions (Fig. 3c) and was more stable at low temperature (< 40 °C), but less stable at medium–high temperature (45–65 °C; Fig. 3d), compared with RSDE from OX*PoxGA15A*. These changes in RSDE properties are potentially very beneficial for industrial applications, such as simultaneous saccharification and fermentation of raw starch to produce bio-ethanol, in which yeasts grow at 30–35 °C [4, 11, 23, 24], using as carbon source, glucose released from raw starch by simultaneous RSDE degradation.

### Hydrolytic analysis of raw starch with crude RSDE produced by mutant A2-13

**Fig. 4:**
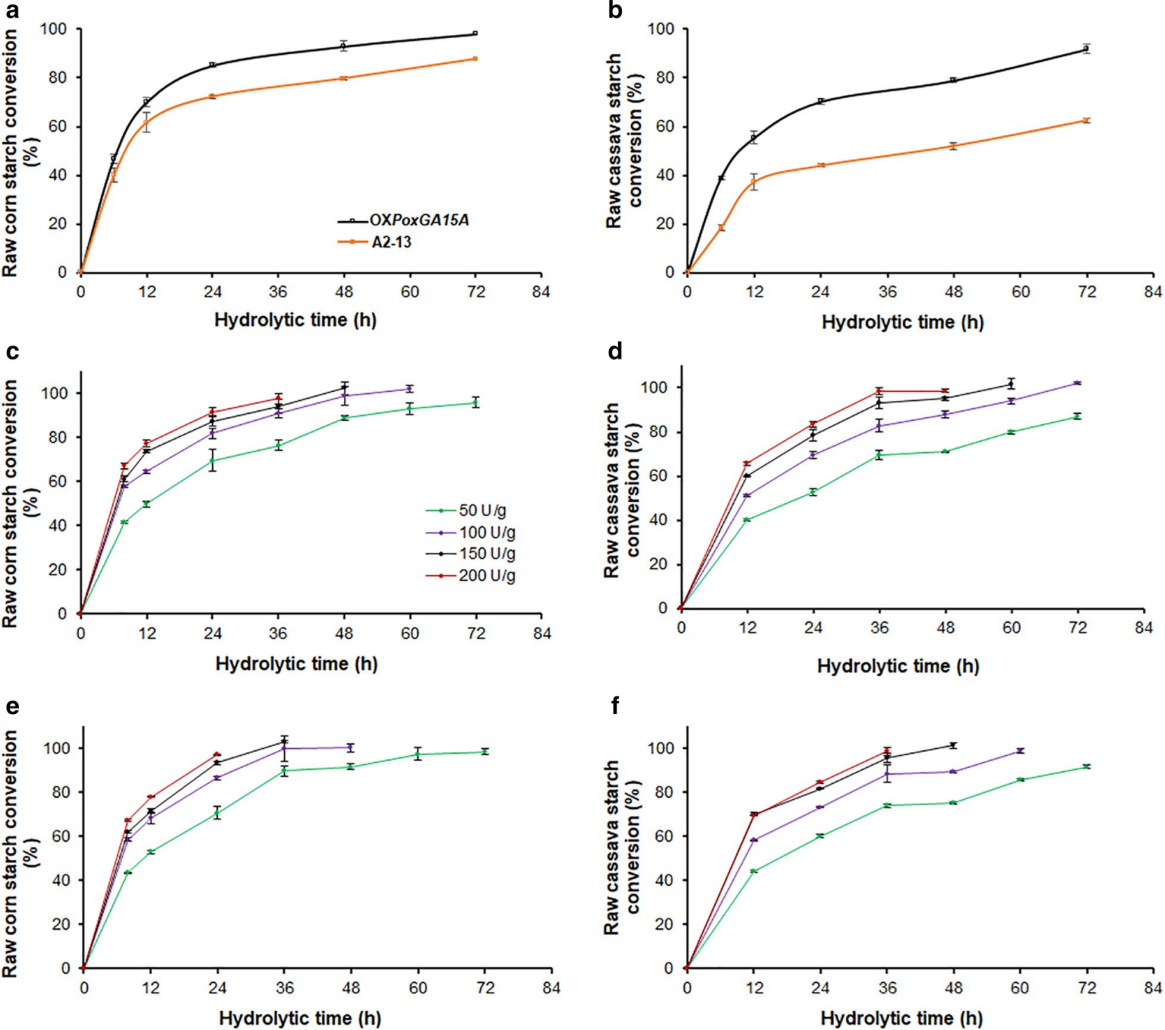
Hydrolysis efficiencies of RNCOF and RNCF by crude enzymes from *P. oxalicum* strains A2-13 and OX*PoxGA15A*. Hydrolysis of RNCOF (**a**) and RNCF (**b**) by crude enzymes from mutants A2-13 and OX*PoxGA15A*. Hydrolysis of RNCOF (**c**) and RNCF (**d**) by crude enzymes from mutant A2-13 combined with commercial α-amylase. Hydrolysis of RNCOF (**e**) and RNCF (**f**) by crude enzymes from OX*PoxGA15A* combined with commercial α-amylase. In panels **a** and **b**, 50 U crude enzyme was added per g of solid substrate. In panels **c**–**f**, the legends show the amounts of crude enzymes from *P. oxalicum* added. The ratio of crude enzymes from *P. oxalicum* and commercial α-amylase was 1:1. Values are presented as the mean ± SD of three replicates for each treatment. *RNCOF* natural raw corn flour, *RNCF* natural raw cassava flour

Corn starch has been widely used as a renewable feedstock for bioethanol production in the US [25]. Cassava starch is readily available in subtropical areas, such as China, Southern Asia, and South Africa, and extensively employed for producing bioethanol and other bio-based products, because of its low cost and non-competition with direct food/feed supplies [3]. Therefore, RNCF and natural raw corn flour (RNCOF) were chosen to evaluate potential applications of A2-13, with OX*PoxGA15A* as control. Analysis of enzymatic hydrolysis reactions revealed that the hydrolytic efficiency of the crude A2-13 enzyme for converting RNCOF into glucose was slightly lower than the OX*PoxGA15A* enzyme (degree of hydrolysis (DH) 87.6% and 98.1% after 72 h, respectively Fig. 4a). In contrast, with RNCF as the substrate, the DHs were 91.8% and 62.5% after 72 h, with the OX*PoxGA15A* and A2-13 enzymes, respectively (Fig. 4b).

When hydrolyzing different substrates using the same enzyme, the substrate composition can contribute to the hydrolytic efficiency. The composition of raw starch granules is mainly amylose and amylopectin, with minor non-starch components, such as cellulose fibers, proteins, and fats [26]. Amylose is made up of long linear polymer chains of glucopyranose units, whereas amylopectin has shorter, but highly branched chains, with high molecular weights [27]. Normally, the amylose content of cassava starch is lower than that of corn starch, but the fiber content is higher [28].

Different DHs from the same substrate, using crude enzymes from strains A2-13 and OX*PoxGA15A*, may result from the two strains producing different RSDE mixtures. Fast degradation of raw starch requires the synergistic action of various enzymes, including α-amylases, glucoamylases, α-glucosidases, and α-1,4-glucan debranching enzymes [7]. For example, the recombinant rPoxGA15A combined with commercial α-amylase efficiently degraded raw corn starch and processed raw cassava flour, but rPoxGA15A alone did not [11].

Therefore, crude enzymes from A2-13 and OX*PoxGA15A* were combined with commercial α-amylase and used to hydrolyze RNCOF and RNCF. Hydrolysis of RNCOF by crude enzyme from A2-13 reached a DH of 96.0% at 72 h, with an enzyme loading of 50 U/g substrate, and a DH of 100% at 60, 48 and 36 h with 100, 150, and 200 U/g substrate, respectively (Fig. 4c). With RNCF as substrate, the DH reached 87.1% at 72 h with an enzyme loading of 50 U/g substrate, and 100% at 72, 60 and 48 h with 100, 150, and 200 U/g (Fig. 4d). The hydrolytic efficiencies against RNCOF (Fig. 4e) and RNCF (Fig. 4f) with crude enzymes from OXPoxGA15A were similar with the enzymes from A2-13, when they were combined with commercial α-amylase.

### Analysis of extracellular proteins and transcriptional levels of major amylase genes in the mutant A2-13

**Fig. 5:**
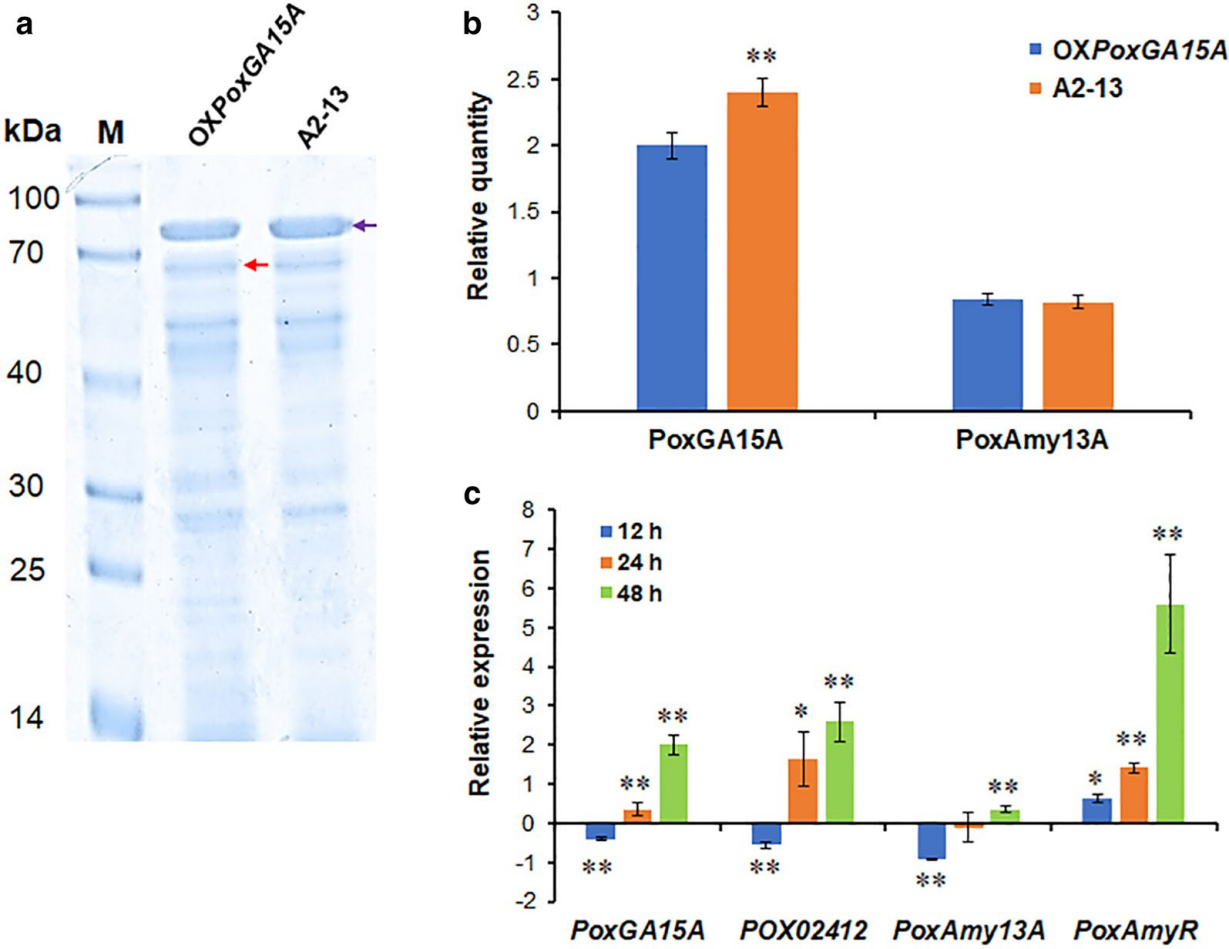
Analysis of extracellular proteins secreted by *P. oxalicum* strains A2-13 and OX*PoxGA15A* (**a**, **b**) and transcription levels of the important amylase genes and their regulatory gene via RT-qPCR analysis (**c**). **a** SDS-PAGE analysis; **b** Relative quantity analysis of the bands in (**a**). In panel **a**, bands marked with purple and red arrows correspond to raw starch-degrading glucoamylase PoxGA15A and α-amylase PoxAmy13A, respectively. In panel **c**, expression levels of the tested genes in the strain A2-13 were normalized against those in the starting strain OX*PoxGA15A*. The actin gene was used as reference. **p ≤ 0.01 and *p ≤ 0.05 indicate significant differences between the A2-13 and OX*PoxGA15A* by Student’s *t* test. Each experiment contained three biological replicates. Each data point represents mean ± SD. *RT-qPCR* real-time reverse transcription quantitative PCR, *SDS-PAGE* sodium dodecyl sulfate polyacrylamide gel electrophoresis, *MMM* modified minimal medium

To better understand the findings from the hydrolysis experiments, the proteins secreted by A2-13 and OX*PoxGA15A,* cultured under their optimal conditions, were compared by SDS-PAGE analysis. The composition of the two protein mixtures was similar, but the relative concentrations of the different components were noticeably different. Specifically, the band corresponding to raw starch-degrading enzyme PoxGA15A [11, 15] in A2-13 was denser than that in OX*PoxGA15A* (Fig. 5a). The PoxGA15A band (labeled by purple arrow in Fig. 5b) in A2-13 was 20% denser than that in OX*PoxGA15A* (*p* < 0.01), but the PoxAmy13A band was not significantly different between A2-13 and OX*PoxGA15A* (Fig. 5). A2-13 apparently produced more raw starch-degrading glucoamylase than OX*PoxGA15A*. Glucoamylase can release glucose from the non-reducing end of starch chains, and acts synergistically with α-amylase, which generates new non-reducing ends in starch chains, thereby improving the hydrolysis efficiency. Moreover, the crude enzymes secreted by A2-13 were a good balance between the different synergistic amylases, which can efficiently digest raw starch.

The expression of the major amylase genes and their regulatory genes in A2-13 on wheat bran plus Avicel was determined by RT-qPCR, i.e., raw starch-degrading glucoamylase gene, *PoxGA15A* (*POX01356*), glucoamylase gene, *POX02412,* as well as α-amylase gene, *PoxAmy13A* (*POX09352*), and its key regulatory gene, *PoxAmyR* (*POX03890)*. After 12 h of culture, transcription of *PoxAmyR* was up-regulated by 62.0%. The other three were down-regulated after 12 h of culture, by 40.8–91.9% (*p* < 0.05), but between 24 and 48 h, they were up-regulated by 33.9–559.5% (*p* < 0.05; Fig. 5c). Modification of amylase gene expression may have resulted from the change in *PoxAmyR* expression.

### Phenotypic and growth analyses of the mutant A2-13

The phenotype and mycelial growth of A2-13 were compared with those of OX*PoxGA15A*. A2-13 was directly inoculated onto solid PDA medium plates containing different carbon sources: wheat bran plus Avicel, Avicel, soluble starch, glucose, and NRCA (RNCF plus Avicel) and cultured for 2‒6 days. The A2-13 colonies on wheat bran plus Avicel, Avicel, PDA, and glucose plates were smaller than those of OX*PoxGA15A* to varying degrees, appeared dense, thick with mycelia, and darker in color. A2-13 colonies on the other carbon sources were darker in color, but not different in size (Additional file 4: Fig. S4A). Sporulation of A2-13 varied on different carbon sources; sporulation was delayed on PDA, wheat bran plus Avicel, and glucose plates, but accelerated on the other carbon sources (Additional file 4: Fig. S4A).

### Genome re-sequencing of the mutant A2-13

To further elucidate the reasons behind the observed changes in enzyme production and phenotypes, genome re-sequencing of A2-13 was carried out, with OX*PoxGA15A* as control. In total, 2881 Mbp of clean data for each *P. oxalicum* strain were generated, with an average sequencing depth of approximately 90-fold, which covered the genome of the wild-type strain HP7-1 with > 99% coverage.

Comparative genomic analyses detected 230 single-nucleotide variations (SNVs) and 131 insertion/deletions (InDels) in the genome of A2-13, compared with that of OX*PoxGA15A*. These SNVs were localized in 193 intergenic regions and 35 coding sequences (CDSs). Of the 35 SNVs in CDSs, 25 non-synonymous SNVs were detected, including one stop, non-synonymous mutation in gene *POX03359* that encodes a hypothetical protein (Additional file 5: Table S1). There were two genes, *POX02883* and *POX04725,* encoding putative transcription factors. Gene *POX09060* encoded the homologous protein of histone methyltransferase Set1, and shared 99.9% identity with PoSet1 (PDE_02489) in *P. oxalicum* strain 114–2 and 64.4% with Set1 in *Saccharomyces cerevisiae*. Set1 performs the methylation of histone H3 lysine 4 (H3K4) [29]. In *S. cerevisiae*, Set1 contributes to assembly of the elongation complex associated with RNA polymerase (Pol) II, by binding nascent RNA [30]. Recently, Li et al. [31] reported that PoSet1 positively regulated the production of cellulases and xylanases in *P. oxalicum,* by modulating H3K4me1 and H3K4me2 signals, as well as regulating colony diameter and spore number. *POX01661* encoded the subunit Tho2 of the THO complex that is required for RNA transcript elongation, mRNA biogenesis, and export in *S*. *cerevisiae* [32, 33]. *POX09438* was annotated as formin Bni1p, which is involved in the polarized localization in *S*. *cerevisiae*, controlled by the Rho GTPase Cdc42, via the effector Gic2p [34]. Cdc42 is activated by the Bud site selection protein Bud3 [35] encoded by *POX05164*. To our surprise, a β-glucosidase gene, *POX03641* was found with a unique SNV and another gene, *POX02958*-encoded calcium/calmodulin-dependent protein kinase type I, and two ribosomal protein-encoding genes, *POX01113* and *POX01133*.

In addition to the SNVs in CDSs, of 193 SNVs located in intergenic regions, 38 and 10 were localized in 1500 bp upstream- and 300 bp downstream regions of the coding genes, respectively (Additional file 5: Table S1). These regions may contain gene promoters and terminators, in which SNVs may regulate the expression of the corresponding genes. A notable finding was a gene, *POX02086,* encoding a zinc finger protein of CCHC-type, two sugar/inositol transporter encoding genes *POX05515* and *POX08241* and an α-glucosidase gene *POX07319*.

InDels in the mutant A2-13 included 65 deletions and 76 insertions. Most of them were located in intergenic regions and introns. Of particular interest, there were six InDels located in four CDSs (*POX00063*, *POX02669*, *POX07393,* and *POX09551*) that encoded endo-β-1,4-xylanase Xyn11A, alkaline phosphatase, ABC transporter, and a hypothetical protein, respectively. InDels in *POX00063* (G deleted at positions 197,194) and *POX09551* (A and G inserted at positions 17,671 and 17,697) resulted in a frameshift mutation in A2-13, whereas deletion of nucleotides in *POX02669* (CTCCCG deleted at position 2,276,066) and *POX07393* (G and TACTCCCC deleted at positions 877 and 878) resulted in removal of a few amino acids at the C-terminus (Additional file 6: Table S2). However, it appears that changes resulting from the InDels in POX00063, POX02669, and POX07393 do not contribute to increased amylase production in A2-13. Elucidation of the biological role of POX09551 requires further study in *P. oxalicum*.

## Conclusions

In this study, we carried out ARTP/EMS-combined mutagenesis to enhance raw starch-degrading enzyme (RSDE) production in *P. oxalicum*. A mutant strain, A2-13, was isolated and produced RSDEs at a concentration of 191.0 U/mL, a yield increase of 89.1% compared with that of the parental strain, OX*PoxGA15A*. Crude RSDE enzymes from A2-13 showed improved tolerance to alkaline conditions and high hydrolysis efficiency against raw starch, when combined with commercial α-amylase. Furthermore, the factors contributing to the improved RSDE production by A2-13 were elucidated. This study confirmed that combined ARTP/EMS is an effective tool to enhance fungal RSDE yields and provided a potential new source of RSDEs for future industrial starch processing.

## Methods

### *P. oxalicum* strains and culture conditions

All *P. oxalicum* strains were cultured on potato-dextrose agar (PDA) plates for spore production. The starting strain, OX*PoxGA15A,* was from the China Center for Type Culture Collection, Wuhan, China (accession number M 2017794; [15]). Spores were collected from *P. oxalicum* cultured on PDA plates for 6 days. Fresh *P. oxalicum* spores (1.0 × 10^8^) were cultured in liquid MMM (g/L: (NH_4_)_2_SO_4_ 4.0, KH_2_PO_4_ 4.0, CaCl_2_ 0.6, MgSO_4_·7H_2_O 0.60, FeSO_4_·7H_2_O 0.005, MnSO_4_ 0.0016, ZnCl_2_ 0.0017, CoCl_2_ 0.002, and 1 mL/L of Tween 80) [36], supplemented with 4% w/v wheat bran, plus 1% w/v Avicel, in an orbital shaker at 180 rpm and 28 °C, for 6 days. The secreted crude enzymes were collected by centrifugation at 11,300×*g* for 15 min and the supernatant used for measurement of enzymatic activity and hydrolysis of natural raw starch flour.

For RT-qPCR analyses, *P. oxalicum* strains (1.0 × 10^8^ spores) were pre-cultured in MMM containing glucose as the sole carbon source for 24 h, then transferred into MMM containing 2% w/v wheat bran, plus 3% w/v Avicel in a shaker, at 180 rpm and 28 °C, for 12–48 h. The mycelia were harvested by filtering for RNA extraction every 12 h.

For genome re-sequencing, *P. oxalicum* strains OX*PoxGA15A* and A2-13 were inoculated into Completed Medium containing (in g/L) yeast extract 1.0, glucose 10.0, casein hydrolysate 1.0 g, NaNO_3_ 6.0, KCl 0.52, MgSO_4_·7H_2_O 0.52, KH_2_PO_4_ 1.52, FeSO_4_·7H_2_O 0.005, MnSO_4_ 0.0016, ZnCl_2_ 0.0017, and CoCl_2_ 0.002, pH 6.5, followed by shaking at 180 rpm and 28 °C, for 48 h.

A two-layer agar gel was prepared, containing raw natural cassava flour (RNCF; from a local farmer’s market in Nanning, China) and ball-milled Avicel (Sigma-Aldrich, Darmstadt, Germany), at a series of different ratios as the top layer, and MMM, without a carbon source as the bottom layer. RNCF was ground directly from freshly harvested cassava tubers after drying, without any other pretreatments, such as cellulose removal.

### Extraction of total DNA and RNA

Total DNA and RNA were extracted from hyphae of *P. oxalicum* strains using chemical methods, as reported previously [36].

### Mutagenesis

For EMS treatment, fresh spores were re-suspended in phosphate buffered saline (PBS) at 10^8^ spores/mL. EMS (1.2%, w/v) was added to the suspension and incubated in a shaker at 180 rpm and 28 °C, for 1–12 h, to determine the optimum treatment time. An equal volume of Na_2_S_2_O_3_ solution was used to stop reaction. The treated spores were separated by centrifugation at 11,300×*g* for 10 min at 4 °C and re-suspended at different concentrations (between 10^2^ and 10^8^/mL) with sterile water.

For ARTP treatment, fresh spores were re-suspended in 5.0% glycerol at 10^6^ spores/mL. Spore suspension (10 µL) was spread on carrier plates and treated for 0 to 550 s at a flow rate of ten liters per minute and radio-frequency power input of 130 W, in a Type M ARTP Mutagenesis Bio-breeding Machine (Wuxi Tmaxtree Biotechnology Co., Ltd., China), to determine the optimum treatment time. The treated spores were washed from the carrier plates using sterile water and adjected to concentrations of 10^2^ to 10^8^ spores/mL.

ARTP/EMS-combined treatment was performed thereafter, as described above, with the optimum treatment times of 8 h by EMS and 500 s by ARTP.

Treated spore suspension (~ 100 μL) was spread on plates containing MMM supplemented with RNCF (1% w/v) plus Avicel (0.5% w/v). After incubation for 8 days at 28 °C, the diameter ratio between each colony and its clear zone was measured and calculated. Spores without EMS and/or ARTP treatment were used as controls.

### Measurement of fungal enzymatic activity

The activity of RSDEs was determined as described previously [37], using RNCF as the substrate. Briefly, crude enzymes from *P. oxalicum* cultures were diluted with citrate buffer (100 mM; pH 4.5). Diluted enzyme solution (50 μL) was added to RNCF (450 μL; 1.0% w/v), incubated at 65 °C for 30 min; then the reaction mixtures were transferred to boiling water for 10 min. Enzymes inactivated by boiling were used as controls. Amounts of released reducing sugars and *p*-nitrophenol were determined using the 3,5-dinitrosalicylic acid method [38] and spectrometry at 410 nm, respectively. One unit of enzymatic activity (U) was defined as the amount of enzyme that produced 1 μmol of reducing sugars per min from each appropriate substrate.

### Optimization of culture conditions

For further enhancement of RSDE production by the mutant A2-13, culture parameters, including the initial medium pH, incubation temperature, carbon source composition, and nitrogen source, were optimized, respectively, using MMM containing wheat bran (4% w/v) plus Avicel (1% w/v). The effect of the initial medium pH on RSDE production by A2-13 was determined at pH 2.5, 3.5, 4.5, 5.5, 6.5, and 7.5, at 28 °C. Subsequently, incubation temperatures of 20, 24, 28, 32, and 36 °C were evaluated at the previously determined optimum pH, to determine the optimum incubation temperature.

Furthermore, at the optimal pH and temperature, MMM containing wheat bran plus other carbon sources (corncob, sugarcane bagasse, rice straw, starch, and soybean cake powder) that replaced Avicel at a ratio of 4:1 (w/w) was assessed for their effects on RSDE production. Subsequently, various ratios of wheat bran to the identified carbon source ([w/w] 1:2; 2:2, 3:2, 1:4, 2:3, 1:3, 3:2, 2:1, and 4:1) were evaluated.

Similarly, several nitrogen sources were evaluated, including soybean cake powder, Urea, NH_4_NO_3_, Peptone, (NH_4_)_2_SO_4_, and NaNO_3_, and the optimum concentration of the best nitrogen source was determined.

Finally, the effects of different-sized inoculations of spores (10^2^, 10^3^, 10^4^, 10^5^, 10^6^, and 10^7^) on RSDE production were determined. All optimization experiments were carried out in a shaking incubator at 180 rpm. *P. oxalicum* was cultured for 6 days and the crude enzymes were collected for RSDE activity measurement.

### Properties of crude RSDE produced by the mutant A2-13

RSDE activity against raw cassava flour (1% w/v) was measured in 0.1 M citrate–phosphate buffer at pH 3.0, 3.5, 4.0, 4.5, 5.0, 5.5, 6.0, 6.5, and 7.0, at 65 °C for 30 min, as described above. RSDE activity at each pH was expressed as relative to that at the optimum pH (designated as 100%). The optimum reaction temperature was then determined at the optimum pH, by measuring the activity at various temperatures (30, 35, 40, 45, 50, 55, 60, 65, 70, 75, and 85 °C). RSDE activity at each temperature was expressed as relative to that at the optimal temperature.

To evaluate pH stability, crude RSDE was incubated at 4 °C for 24 h in buffers at various pHs, i.e., 0.1 M citrate-Na_2_HPO_4_ buffer (pH 3.0–7.0), Tris–HCl buffer (pH 7.0–9.0), and glycine–NaOH buffer (pH 9.0–11.0); then the residual RSDE activity was determined at the optimum pH and temperature for activity. Activity of untreated RSDE was defined as 100% and used as the positive control. Similarly, to ascertain temperature stability, crude RSDE was added to buffer at the optimum pH for stability, and the mixture was incubated at various temperatures from 30 to 65 °C, for 1 h. The residual RSDE activity was calculated as described above.

### Enzymatic hydrolysis of raw starch

RNCF and natural raw corn flour (RNCOF, purchased from a farmer’s market in Nanning, China) were used as substrates for enzymatic hydrolysis. For raw starch hydrolysis by RSDEs from *P. oxalicum* A2-13 and OX*PoxGA15A*, hydrolysis reactions with 10% (w/v) solid substrate and an enzyme loading of 50 U/g substrate were conducted in citrate–phosphate buffer (pH 4.5) at 40 °C for 72 h. The reducing sugars produced were determined using the DNS method [38].

A combination of RSDEs from the *P. oxalicum* strains with commercial α-amylase (Solarbio, Beijing, China) at a ratio of 1:1 was also tested, as described above. RSDEs were added at several concentrations ([U/g substrate], 50, 100, 150, and 200).

### Genome re-sequencing

Genomic DNAs of *P. oxalicum* strains A2-13 and OX*PoxGA15A* were extracted and subsequently used for construction of the read libraries with a length of 400 bp. Libraries were sequenced on a BGISEQ-500 platform at the Beijing Genomics Institute (BGI, Shenzhen, China). After removing low-quality reads and adaptors, the generated clean reads were mapped onto the genome of *P. oxalicum* strain HP7-1 [16] using Bowtie2 v 0.7.10 [39] to detect SNVs.

### RT-qPCR assay

RT-qPCR was carried out as described previously [16]. The tested genes were detected using specific primers (Additional file 7: Table S3). The actin gene *POX09428* was used as reference. The expression level of each tested gene was calculated relative to that of *POX09428*, and subsequently normalized against that for the parental strain Δ*PoxKu70*. Each experiment was repeated at least in triplicate.

### Analysis by sodium dodecyl sulfate polyacrylamide gel electrophoresis

Proteins secreted by *P. oxalicum* strains OX*PoxGA15A* and A2-13 cultured in MMM, containing wheat bran plus Avicel, for 6 days, were analyzed using sodium dodecyl sulfate polyacrylamide gel electrophoresis (SDS-PAGE). The relative quantity of each protein was measured by GS-900TM calibrated densitometer (Bio-Rad, Hercules, CA, USA) with a software ImageLab.

### Light microcopy

A Canon EOS 6D digital camera (Canon, Beijing, China) was used to photograph *P. oxalicum* colonies on agar plates. In addition, harvested mycelia were transferred to microscope slides, and photographed using an Olympus DP480 microscope (Olympus Corporation, Tokyo, Japan). Photomicrographs were analyzed using cellSens Dimension digital imaging software (Olympus).

### Statistical analysis

Statistical analysis was performed using Microsoft Excel (Office 2016; Microsoft, Redmond, WA) by Student’s *t* test.

### Accession number

Re-sequenced data are available from the Sequence Read Archive database under accession number SRA493765.

## Supplementary information

**Additional file 1: Fig. S1.** ARTP/EMS-mediated mutagenesis and screening for RSDE hyperproducers. The *P. oxalicum* isolates were grown on the two-layer agar gel plates for 8 days. *: the isolates were screened and used for the next round of mutagenesis, by comparative analysis of their RSDE activities in MMM, containing wheat bran plus Avicel as the carbon source, for 6 days. RSDE activity was determined using RNCF as the substrate. ARTP: atmospheric and room-temperature plasma; EMS: ethyl methyl sulfonate; RNCF: natural raw cassava flour; MMM: modified minimal medium.

**Additional file 2: Fig. S2.** Curve showing lethality against *P. oxalicum* strains OX*PoxGA15A* and E3-16, treated by ethyl methyl sulfonate (A) and atmospheric and room-temperature plasma (B). *P. oxalicum* spores were spread on PDA plates and incubated at 28 °C for 4 days. Each data point represents mean ± SD. Each experiment contained three biological replicates.

**Additional file 3: Fig. S3.** Effects of culture conditions and inoculated spore number on RSDE production of *P. oxalicum* strain A2-13. (A) Initial pH of medium; (B) incubation temperature; (C) Carbon source; (D) Proportions of wheat bran and Avicel; (E) Nitrogen source; (F) NH_4_NO_3_ concentration; (G) inoculated spore number. *P. oxalicum* strains were cultured at 28 °C and 180 rpm for 6 days. Each data point represents mean ± SD. Each experiment contained three biological replicates.

**Additional file 4: Fig. S4.** Colonic (A) and mycelial (B) analysis of the *P. oxalicum* strains A2-13 and OX*PoxGA15A* on plates containing various carbon sources. RNCA: natural raw cassava flour plus Avicel, RNCF: natural raw cassava flour, PDA: potato dextrose agar. Scale bar = 100 μm.

**Additional file 5: Table S1.** List of 230 single-nucleotide variants of the mutant A2-13 compared with the starting strain OX*PoxGA15A*.

** Additional file 6: Table S2. **Mutation sites in coding sequences (CDS) in the mutant A2-13 compared with that in OX*PoxGA15A*.

**Additional file 7: Table S3. **Primers used in this study.

## Data Availability

Re-sequenced data are available from the Sequence Read Archive database under Accession number SRA493765.

## Abbreviations

ARTP: Atmospheric and room-temperature plasma
EMS: Ethyl methanesulfonate
RT-qPCR: Real-time reverse transcription quantitative PCR
SNP: Single-nucleotide polymorphism
SNVs: Single-nucleotide variations
SDS-PAGE: Sodium dodecyl sulfate polyacrylamide gel electrophoresis
RSDE: Raw starch-degrading enzyme
MMM: Modified minimal medium
RNCF: Natural raw cassava flour
RNCOF: Natural raw corn flour
NRCA: NRCF plus Avicel
