# Supplementary material for: ARTP/EMS-combined multiple mutagenesis efficiently improved production of raw starch-degrading enzymes in Penicillium oxalicum and characterization of the enzyme-hyperproducing mutant
Source: Biotechnol Biofuels. 2020 Nov 11;13:187. doi: 10.1186/s13068-020-01826-5 (PMC7661180; doi:10.1186/s13068-020-01826-5)
Supplement: Supplementary file 1 — Additional file 1: Fig. S1. ARTP/EMS-mediated mutagenesis and screening for RSDE hyperproducers. The P. oxalicum isolates were grown on the two-layer agar gel plates for 8 days. *: the isolates were screened and used for the next round of mutagenesis, by comparative analysis of their RSDE activities in MMM, containing wheat bran plus Avicel as the carbon source, for 6 days. RSDE activity was determined using RNCF as the substrate. ARTP: atmospheric and room-temperature plasma; EMS: ethyl methyl sulfonate; RNCF: natural raw cassava flour; MMM: modified minimal medium. [file 13068_2020_1826_MOESM1_ESM.pdf]

| Mutagenesis steps | Number of colonies isolated | Number of isolates for next round of mutagenesis* | Threshold for diameter ratio between clearing zone and colony |
|---|---|---|---|
| Start strain *OXPoxGA15A* | | | |
| ↓ 1st EMS mutagenesis | 441 | 13 | > 1.48 |
| Mutant E1-1 | | | |
| ↓ 2nd EMS mutagenesis | 352 | 5 | > 1.50 |
| Mutant E2-3 | | | |
| ↓ 3rd EMS mutagenesis | 823 | 20 | > 1.68 |
| Mutant E3-16 | | | |
| ↓ ARTP mutagenesis | 1120 | 35 | > 1.70 |
| Mutant A1-2 | | | |
| ↓ ARTP-EMS mutagenesis | 796 | 28 | > 1.75 |
| Mutant A2-13 | | | |
