# Supplementary figures and images for: ARTP/EMS-combined multiple mutagenesis efficiently improved production of raw starch-degrading enzymes in Penicillium oxalicum and characterization of the enzyme-hyperproducing mutant
Source: Biotechnol Biofuels. 2020 Nov 11;13:187. doi: 10.1186/s13068-020-01826-5 (PMC7661180; doi:10.1186/s13068-020-01826-5)

**A**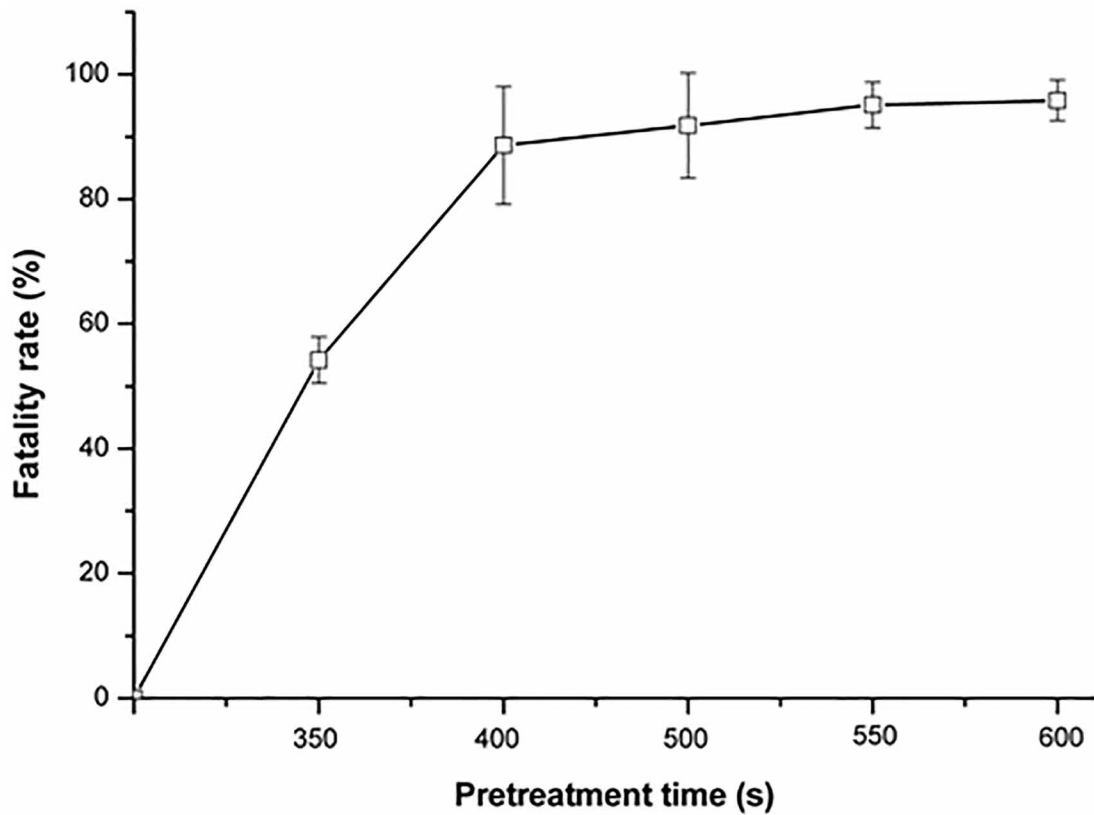**B**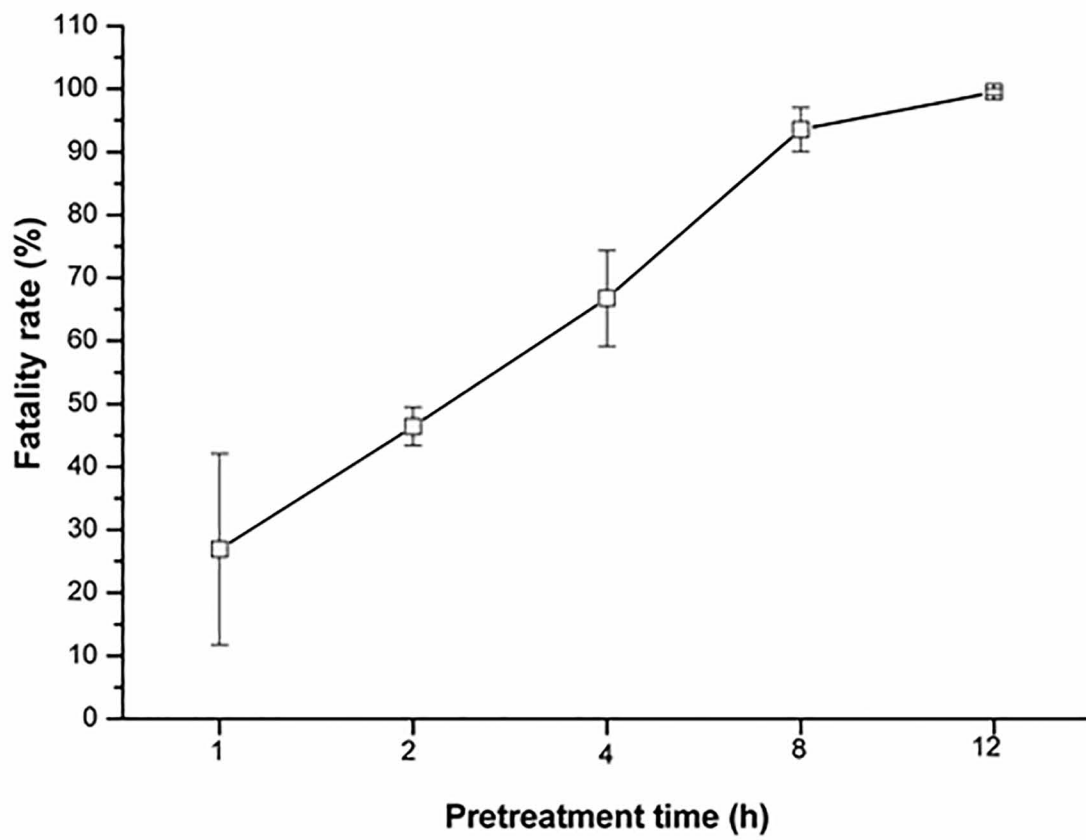

Supplement: Supplementary file 2 — Additional file 2: Fig. S2. Curve showing lethality against P. oxalicum strains OXPoxGA15A and E3-16, treated by ethyl methyl sulfonate (A) and atmospheric and room-temperature plasma (B). P. oxalicum spores were spread on PDA plates and incubated at 28 °C for 4 days. Each data point represents mean ± SD. Each experiment contained three biological replicates. [file 13068_2020_1826_MOESM2_ESM.pdf]

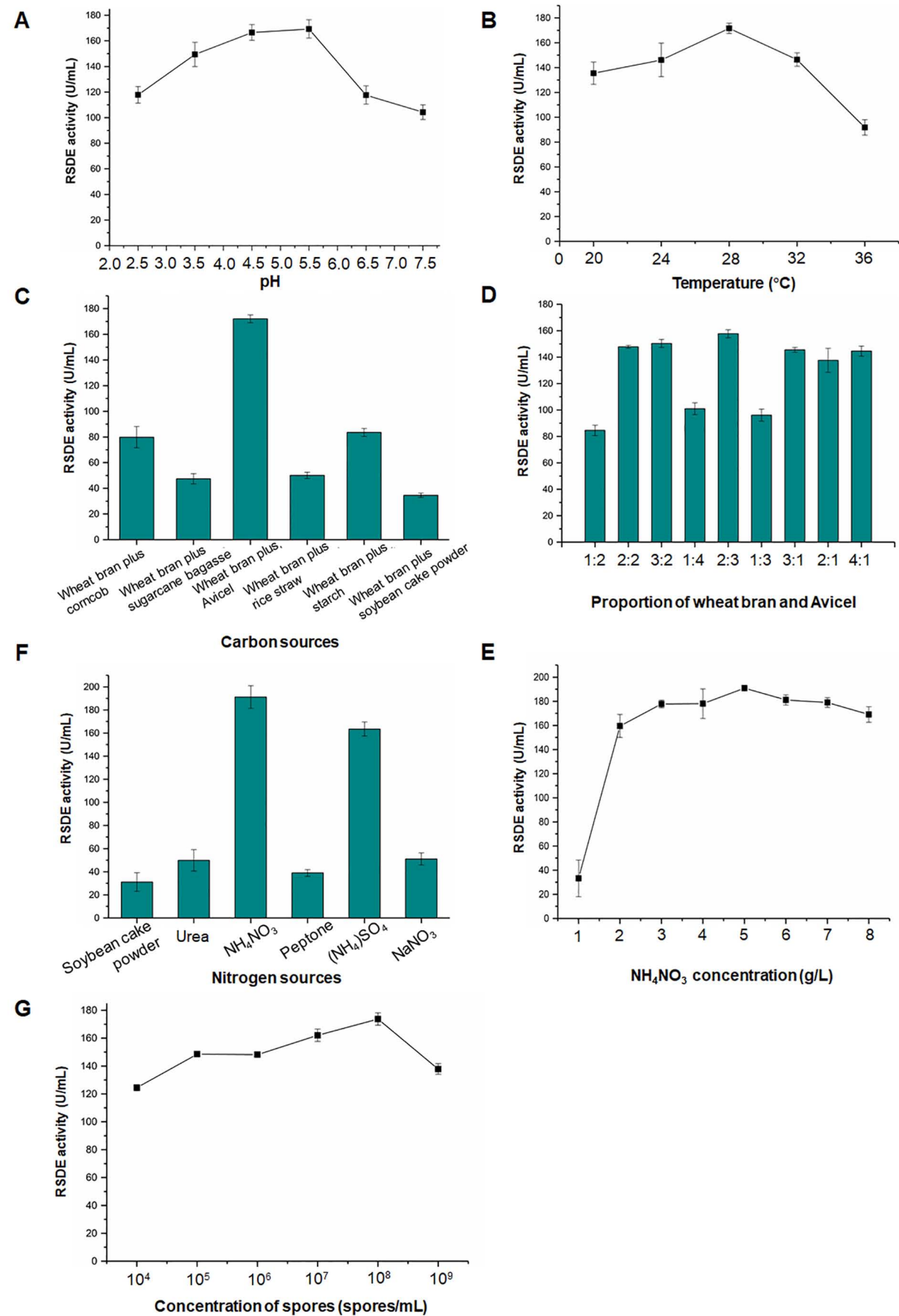

Supplement: Supplementary file 3 — Additional file 3: Fig. S3. Effects of culture conditions and inoculated spore number on RSDE production of P. oxalicum strain A2-13. (A) Initial pH of medium; (B) incubation temperature; (C) Carbon source; (D) Proportions of wheat bran and Avicel; (E) Nitrogen source; (F) NH4NO3 concentration; (G) inoculated spore number. P. oxalicum strains were cultured at 28 °C and 180 rpm for 6 days. Each data point represents mean ± SD. Each experiment contained three biological replicates. [file 13068_2020_1826_MOESM3_ESM.pdf]

**A**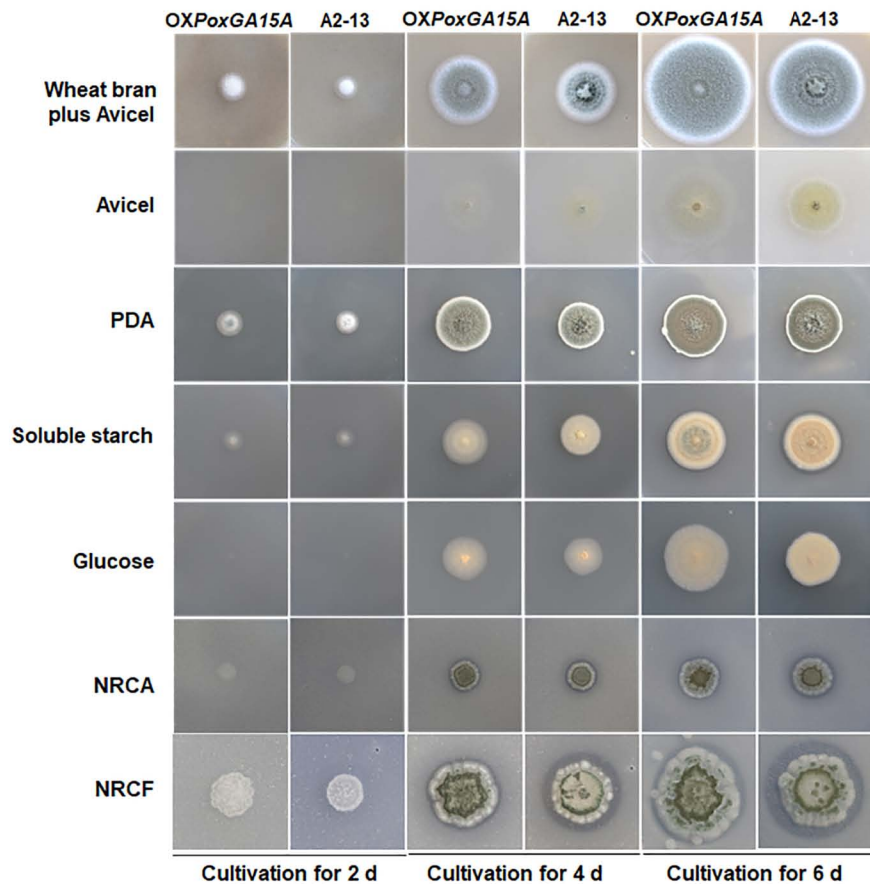**B**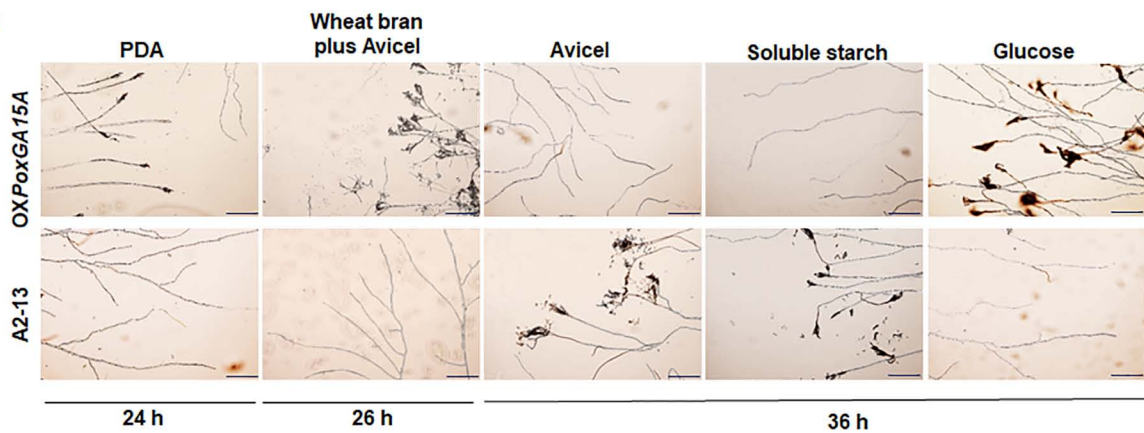

Supplement: Supplementary file 4 — Additional file 4: Fig. S4. Colonic (A) and mycelial (B) analysis of the P. oxalicum strains A2-13 and OXPoxGA15A on plates containing various carbon sources. RNCA: natural raw cassava flour plus Avicel, RNCF: natural raw cassava flour, PDA: potato dextrose agar. Scale bar = 100 μm. [file 13068_2020_1826_MOESM4_ESM.pdf]
