# Supplementary material for: ARTP/EMS-combined multiple mutagenesis efficiently improved production of raw starch-degrading enzymes in Penicillium oxalicum and characterization of the enzyme-hyperproducing mutant
Source: Biotechnol Biofuels. 2020 Nov 11;13:187. doi: 10.1186/s13068-020-01826-5 (PMC7661180; doi:10.1186/s13068-020-01826-5)
Supplement: Supplementary file 5 — Additional file 5: Table S1. List of 230 single-nucleotide variants of the mutant A2-13 compared with the starting strain OXPoxGA15A. [file 13068_2020_1826_MOESM5_ESM.pdf]

Additional file 5: Table S1. List of 230 single nucleotide variants in the mutant A2-13 compared with the start strain OX*PoxGA15A*

		A2-13				A2-13						
**Gene ID**	**Location**	**Referenc**	**Codon muta**	**AA mutate**	**Mutate type**	**Location in s**	**Position**	**Reference**	**Sample ba**	**Gene dista**	**Location**	**Protein function**
POX00419		NA	NA	NA	NA	scaffold_1	1308266	C	T	1915	Intergenic region	Hypothetical protein
POX01041		NA	NA	NA	NA	scaffold_1	3168919	A	G	4025	Intergenic region	Hypothetical protein
POX01041		NA	NA	NA	NA	scaffold_1	3184639	G	A	19745	Intergenic region	Hypothetical protein
POX01042		NA	NA	NA	NA	scaffold_1	3194726	C	T	28908	Intergenic region	Hypothetical protein
POX01042		NA	NA	NA	NA	scaffold_1	3198496	T	C	25138	Intergenic region	Hypothetical protein
POX01042		NA	NA	NA	NA	scaffold_1	3205591	T	C	18043	Intergenic region	Hypothetical protein
POX01042		NA	NA	NA	NA	scaffold_1	3205773	T	A	17861	Intergenic region	Hypothetical protein
POX01042		NA	NA	NA	NA	scaffold_1	3205803	T	C	17831	Intergenic region	Hypothetical protein
POX01042		NA	NA	NA	NA	scaffold_1	3220008	C	T	3626	Intergenic region	Hypothetical protein
POX01042		NA	NA	NA	NA	scaffold_1	3220269	C	T	3365	Intergenic region	Hypothetical protein
POX01042		NA	NA	NA	NA	scaffold_1	3221831	G	A	1803	Intergenic region	Hypothetical protein
POX01044		NA	NA	NA	NA	scaffold_1	3231928	A	G	3194	Intergenic region	Hypothetical protein
POX01048		NA	NA	NA	NA	scaffold_1	3259082	G	A	12376	Intergenic region	Putative surface membrane protein
POX01316		NA	NA	NA	NA	scaffold_1	4052600	C	T	1440	Intergenic region	Hypothetical protein
POX01587		NA	NA	NA	NA	scaffold_1	4846050	G	A	440	Intergenic region	Hypothetical protein
POX02020		NA	NA	NA	NA	scaffold_2	278039	A	G	386	Intergenic region	Hypothetical protein
POX02133		NA	NA	NA	NA	scaffold_2	647264	T	C	5647	Intergenic region	Hypothetical protein
POX02134		NA	NA	NA	NA	scaffold_2	712930	T	A	6347	Intergenic region	Hypothetical protein
POX02479		NA	NA	NA	NA	scaffold_2	1705922	C	T	1150	Intergenic region	Hypothetical protein
POX02596		NA	NA	NA	NA	scaffold_2	2056183	A	G	637	Intergenic region	Hypothetical protein
POX02676		NA	NA	NA	NA	scaffold_2	2294921	G	A	832	Intergenic region	Hypothetical protein
POX02938		NA	NA	NA	NA	scaffold_2	3082590	A	G	574	Intergenic region	Hypothetical protein
POX02949		NA	NA	NA	NA	scaffold_2	3134389	C	T	1304	Intergenic region	Hypothetical protein
POX03236		NA	NA	NA	NA	scaffold_2	4016405	G	A	4978	Intergenic region	Zn2Cys6
POX03417		NA	NA	NA	NA	scaffold_2	4531372	G	A	314	Intergenic region	Hypothetical protein
POX03810		NA	NA	NA	NA	scaffold_3	1082168	C	T	2754	Intergenic region	Hypothetical protein
POX03821		NA	NA	NA	NA	scaffold_3	1110547	G	A	966	Intergenic region	Hypothetical protein
POX03875		NA	NA	NA	NA	scaffold_3	1279965	A	T	10685	Intergenic region	Hypothetical protein
POX03875		NA	NA	NA	NA	scaffold_3	1282287	A	G	13007	Intergenic region	Hypothetical protein
POX03875		NA	NA	NA	NA	scaffold_3	1287904	A	C	18624	Intergenic region	Hypothetical protein
POX03875		NA	NA	NA	NA	scaffold_3	1295492	A	G	26212	Intergenic region	Hypothetical protein
POX03875		NA	NA	NA	NA	scaffold_3	1315727	T	C	46447	Intergenic region	Hypothetical protein
POX03876		NA	NA	NA	NA	scaffold_3	1317965	A	G	45311	Intergenic region	Hypothetical protein
POX03876		NA	NA	NA	NA	scaffold_3	1318363	A	G	44913	Intergenic region	Hypothetical protein
POX03876		NA	NA	NA	NA	scaffold_3	1341087	C	T	22189	Intergenic region	Hypothetical protein
POX03876		NA	NA	NA	NA	scaffold_3	1358384	A	G	4892	Intergenic region	Hypothetical protein
POX03876		NA	NA	NA	NA	scaffold_3	1358400	G	A	4876	Intergenic region	Hypothetical protein
POX03876		NA	NA	NA	NA	scaffold_3	1358427	T	C	4849	Intergenic region	Hypothetical protein
POX03876		NA	NA	NA	NA	scaffold_3	1362485	T	C	791	Intergenic region	Hypothetical protein
POX03876		NA	NA	NA	NA	scaffold_3	1362906	T	C	370	Intergenic region	Hypothetical protein
POX04008		NA	NA	NA	NA	scaffold_3	1783360	G	A	1632	Intergenic region	Hypothetical protein
POX04538		NA	NA	NA	NA	scaffold_4	581	A	G	11899	Intergenic region	Hypothetical protein
POX04806		NA	NA	NA	NA	scaffold_4	819744	C	T	475	Intergenic region	Hypothetical protein
POX05062		NA	NA	NA	NA	scaffold_4	1530788	A	T	3200	Intergenic region	Hypothetical protein
POX05667		NA	NA	NA	NA	scaffold_5	19949	C	G	536	Intergenic region	Hypothetical protein
POX05904		NA	NA	NA	NA	scaffold_5	766324	A	T	1584	Intergenic region	Hypothetical protein
POX06196		NA	NA	NA	NA	scaffold_5	1702039	C	T	526	Intergenic region	Hypothetical protein
POX06663		NA	NA	NA	NA	scaffold_6	185528	T	G	1132	Intergenic region	Hypothetical protein
POX06667		NA	NA	NA	NA	scaffold_6	198239	A	G	2048	Intergenic region	Hypothetical protein
POX06667		NA	NA	NA	NA	scaffold_6	198278	T	C	2087	Intergenic region	Hypothetical protein
POX06667		NA	NA	NA	NA	scaffold_6	198881	T	C	2690	Intergenic region	Hypothetical protein
POX06667		NA	NA	NA	NA	scaffold_6	200285	G	A	4094	Intergenic region	Hypothetical protein
POX06667		NA	NA	NA	NA	scaffold_6	200353	A	G	4162	Intergenic region	Hypothetical protein
POX06668		NA	NA	NA	NA	scaffold_6	229405	A	G	6811	Intergenic region	Hypothetical protein

POX06668		NA	NA	NA	NA	scaffold_6	229420	C	T	6826	Intergenic region	Hypothetical protein
POX06668		NA	NA	NA	NA	scaffold_6	232334	A	G	9740	Intergenic region	Hypothetical protein
POX06669		NA	NA	NA	NA	scaffold_6	243337	C	T	3880	Intergenic region	Hypothetical protein
POX06671		NA	NA	NA	NA	scaffold_6	274123	T	C	17279	Intergenic region	Hypothetical protein
POX06671		NA	NA	NA	NA	scaffold_6	275350	C	G	18506	Intergenic region	Hypothetical protein
POX06671		NA	NA	NA	NA	scaffold_6	275395	A	G	18551	Intergenic region	Hypothetical protein
POX06671		NA	NA	NA	NA	scaffold_6	275514	T	C	18670	Intergenic region	Hypothetical protein
POX06671		NA	NA	NA	NA	scaffold_6	275773	G	A	18929	Intergenic region	Hypothetical protein
POX06672		NA	NA	NA	NA	scaffold_6	293950	T	C	4020	Intergenic region	Hypothetical protein
POX06672		NA	NA	NA	NA	scaffold_6	295148	C	T	2822	Intergenic region	Hypothetical protein
POX06672		NA	NA	NA	NA	scaffold_6	299104	G	A	733	Intergenic region	Hypothetical protein
POX06674		NA	NA	NA	NA	scaffold_6	302763	A	G	657	Intergenic region	Hypothetical protein
POX06675		NA	NA	NA	NA	scaffold_6	313512	C	T	6290	Intergenic region	Hypothetical protein
POX06675		NA	NA	NA	NA	scaffold_6	313619	T	C	6183	Intergenic region	Hypothetical protein
POX06675		NA	NA	NA	NA	scaffold_6	313725	T	C	6077	Intergenic region	Hypothetical protein
POX06675		NA	NA	NA	NA	scaffold_6	313872	C	T	5930	Intergenic region	Hypothetical protein
POX07714		NA	NA	NA	NA	scaffold_7	1021897	G	A	3163	Intergenic region	Hypothetical protein
POX07714		NA	NA	NA	NA	scaffold_7	1022751	T	C	2309	Intergenic region	Hypothetical protein
POX07714		NA	NA	NA	NA	scaffold_7	1022863	T	C	2197	Intergenic region	Hypothetical protein
POX07717		NA	NA	NA	NA	scaffold_7	1066243	A	G	509	Intergenic region	Hypothetical protein
POX07717		NA	NA	NA	NA	scaffold_7	1066674	C	T	940	Intergenic region	Hypothetical protein
POX07717		NA	NA	NA	NA	scaffold_7	1070896	A	G	1721	Intergenic region	Hypothetical protein
POX07717		NA	NA	NA	NA	scaffold_7	1073555	C	G	5162	Intergenic region	Hypothetical protein
POX07793		NA	NA	NA	NA	scaffold_7	1321733	A	C	1104	Intergenic region	Hypothetical protein
POX07874		NA	NA	NA	NA	scaffold_7	1629840	C	T	4158	Intergenic region	Hypothetical protein
POX07920		NA	NA	NA	NA	scaffold_7	1763852	G	A	1464	Intergenic region	Hypothetical protein
POX08273		NA	NA	NA	NA	scaffold_8	994427	C	T	2928	Intergenic region	Hypothetical protein
POX08273		NA	NA	NA	NA	scaffold_8	998272	G	A	6773	Intergenic region	Hypothetical protein
POX08273		NA	NA	NA	NA	scaffold_8	998336	A	G	6837	Intergenic region	Hypothetical protein
POX08273		NA	NA	NA	NA	scaffold_8	998398	G	A	6899	Intergenic region	Hypothetical protein
POX08274		NA	NA	NA	NA	scaffold_8	1012819	G	A	2016	Intergenic region	Hypothetical protein
POX08344		NA	NA	NA	NA	scaffold_8	1207151	G	A	1895	Intergenic region	Hypothetical protein
POX08500		NA	NA	NA	NA	scaffold_9	18256	T	C	9062	Intergenic region	Centromere protein B, DNA-binding region
POX08500		NA	NA	NA	NA	scaffold_9	23086	T	C	13892	Intergenic region	Centromere protein B, DNA-binding region
POX08500		NA	NA	NA	NA	scaffold_9	23881	T	C	14687	Intergenic region	Centromere protein B, DNA-binding region
POX08500		NA	NA	NA	NA	scaffold_9	24213	T	C	15019	Intergenic region	Centromere protein B, DNA-binding region
POX08500		NA	NA	NA	NA	scaffold_9	24238	C	T	15044	Intergenic region	Centromere protein B, DNA-binding region
POX08500		NA	NA	NA	NA	scaffold_9	24273	T	C	15079	Intergenic region	Centromere protein B, DNA-binding region
POX08501		NA	NA	NA	NA	scaffold_9	35401	A	G	12504	Intergenic region	Hypothetical protein
POX08532		NA	NA	NA	NA	scaffold_9	134484	C	T	324	Intergenic region	Hypothetical protein
POX08694		NA	NA	NA	NA	scaffold_9	652657	T	G	5501	Intergenic region	Hypothetical protein
POX08703		NA	NA	NA	NA	scaffold_9	692824	A	G	5031	Intergenic region	Hypothetical protein
POX09308		NA	NA	NA	NA	scaffold_13	30124	T	C	5940	Intergenic region	Putative trehalose phosphorylase
POX09308		NA	NA	NA	NA	scaffold_13	30141	T	C	5957	Intergenic region	Putative trehalose phosphorylase
POX09308		NA	NA	NA	NA	scaffold_13	30165	G	A	5981	Intergenic region	Putative trehalose phosphorylase
POX09309		NA	NA	NA	NA	scaffold_13	43557	A	G	1118	Intergenic region	Hypothetical protein
POX09309		NA	NA	NA	NA	scaffold_13	43574	C	T	1101	Intergenic region	Hypothetical protein
POX09309		NA	NA	NA	NA	scaffold_13	43607	T	C	1068	Intergenic region	Hypothetical protein
POX09545		NA	NA	NA	NA	scaffold_14	283354	T	C	3551	Intergenic region	Hypothetical protein
POX09545		NA	NA	NA	NA	scaffold_14	299327	A	G	19524	Intergenic region	Hypothetical protein
POX09545		NA	NA	NA	NA	scaffold_14	315108	T	C	35305	Intergenic region	Hypothetical protein
POX09545		NA	NA	NA	NA	scaffold_14	315271	A	G	35468	Intergenic region	Hypothetical protein
POX09545		NA	NA	NA	NA	scaffold_14	315297	T	C	35494	Intergenic region	Hypothetical protein
POX09545		NA	NA	NA	NA	scaffold_14	316919	C	A	37116	Intergenic region	Hypothetical protein
POX09545		NA	NA	NA	NA	scaffold_14	316970	C	T	37167	Intergenic region	Hypothetical protein
POX09552		NA	NA	NA	NA	scaffold_15	24028	C	T	1341	Intergenic region	Hypothetical protein
POX09559		NA	NA	NA	NA	scaffold_15	48491	A	C	1992	Intergenic region	Hypothetical protein

POX09628		NA	NA	NA	NA	scaffold_16	1262	A	G	747	Intergenic region	Hypothetical protein
POX09630		NA	NA	NA	NA	scaffold_16	7913	T	C	2798	Intergenic region	Hypothetical protein
POX09631		NA	NA	NA	NA	scaffold_16	12955	C	T	5645	Intergenic region	Hypothetical protein
POX09678		NA	NA	NA	NA	scaffold_16	156245	C	T	5120	Intergenic region	Hypothetical protein
POX09678		NA	NA	NA	NA	scaffold_16	157493	C	T	6368	Intergenic region	Hypothetical protein
POX09678		NA	NA	NA	NA	scaffold_16	157505	G	A	6380	Intergenic region	Hypothetical protein
POX09678		NA	NA	NA	NA	scaffold_16	157547	G	A	6422	Intergenic region	Hypothetical protein
POX09679		NA	NA	NA	NA	scaffold_16	182948	G	A	4378	Intergenic region	Hypothetical protein
POX09679		NA	NA	NA	NA	scaffold_16	182968	A	G	4358	Intergenic region	Hypothetical protein
POX09679		NA	NA	NA	NA	scaffold_16	185777	C	T	1549	Intergenic region	Hypothetical protein
POX09683		NA	NA	NA	NA	scaffold_16	205554	A	G	8153	Intergenic region	Hypothetical protein
POX09683		NA	NA	NA	NA	scaffold_16	208907	G	A	4800	Intergenic region	Hypothetical protein
POX09740		NA	NA	NA	NA	scaffold_17	216677	A	G	2367	Intergenic region	Hypothetical protein
POX09745		NA	NA	NA	NA	scaffold_18	20986	G	A	9204	Intergenic region	Hypothetical protein
POX09745		NA	NA	NA	NA	scaffold_18	21114	C	T	9076	Intergenic region	Hypothetical protein
POX09745		NA	NA	NA	NA	scaffold_18	21155	G	A	9035	Intergenic region	Hypothetical protein
POX09745		NA	NA	NA	NA	scaffold_18	21255	C	T	8935	Intergenic region	Hypothetical protein
POX09746		NA	NA	NA	NA	scaffold_18	57351	C	T	25361	Intergenic region	Hypothetical protein
POX09746		NA	NA	NA	NA	scaffold_18	57915	G	T	24797	Intergenic region	Hypothetical protein
POX09746		NA	NA	NA	NA	scaffold_18	57942	G	A	24770	Intergenic region	Hypothetical protein
POX09746		NA	NA	NA	NA	scaffold_18	62205	A	G	20507	Intergenic region	Hypothetical protein
POX09782		NA	NA	NA	NA	scaffold_19	31053	C	T	1562	Intergenic region	Hypothetical protein
POX09799		NA	NA	NA	NA	scaffold_20	343	C	T	8266	Intergenic region	Hypothetical protein
POX09800		NA	NA	NA	NA	scaffold_20	18904	G	A	4466	Intergenic region	Centromere protein B, DNA-binding region
POX09800		NA	NA	NA	NA	scaffold_20	18929	T	C	4491	Intergenic region	Centromere protein B, DNA-binding region
POX09803		NA	NA	NA	NA	scaffold_21	5079	A	G	14270	Intergenic region	Hypothetical protein
POX09803		NA	NA	NA	NA	scaffold_21	8515	A	G	10834	Intergenic region	Hypothetical protein
POX09804		NA	NA	NA	NA	scaffold_21	31625	G	A	1903	Intergenic region	Hypothetical protein
POX09804		NA	NA	NA	NA	scaffold_21	31638	G	A	1916	Intergenic region	Hypothetical protein
POX09804		NA	NA	NA	NA	scaffold_21	31777	T	C	2055	Intergenic region	Hypothetical protein
POX09804		NA	NA	NA	NA	scaffold_21	31824	G	A	2101	Intergenic region	Hypothetical protein
POX09804		NA	NA	NA	NA	scaffold_21	31835	A	G	2113	Intergenic region	Hypothetical protein
POX09804		NA	NA	NA	NA	scaffold_21	35508	T	C	5786	Intergenic region	Hypothetical protein
POX09804		NA	NA	NA	NA	scaffold_21	35648	G	A	5926	Intergenic region	Hypothetical protein
POX00489		NA	NA	NA	NA	scaffold_1	1511639	T	C	205	1500 bp unstream region	Hypothetical protein
POX00835		NA	NA	NA	NA	scaffold_1	2505008	T	C	152	1500 bp unstream region	Hypothetical protein
POX00916		NA	NA	NA	NA	scaffold_1	2767275	G	A	612	1500 bp unstream region	Hypothetical protein
POX01043		NA	NA	NA	NA	scaffold_1	3227729	C	T	496	1500 bp unstream region	Hypothetical protein
POX01152		NA	NA	NA	NA	scaffold_1	3592567	A	G	1440	1500 bp unstream region	Hypothetical protein
POX01285		NA	NA	NA	NA	scaffold_1	3946990	G	A	472	1500 bp unstream region	Hypothetical protein
POX01487		NA	NA	NA	NA	scaffold_1	4556201	T	A	102	1500 bp unstream region	Hypothetical protein
POX01513		NA	NA	NA	NA	scaffold_1	4622888	C	T	1446	1500 bp unstream region	Hypothetical protein
POX02086		NA	NA	NA	NA	scaffold_2	475426	G	A	565	1500 bp unstream region	Zinc finger, CCHC-type
POX02086		NA	NA	NA	NA	scaffold_2	475437	T	C	554	1500 bp unstream region	Zinc finger, CCHC-type
POX03280		NA	NA	NA	NA	scaffold_2	4144696	G	A	320	1500 bp unstream region	Hypothetical protein
POX03761		NA	NA	NA	NA	scaffold_3	850869	C	G	337	1500 bp unstream region	Hypothetical protein
POX03761		NA	NA	NA	NA	scaffold_3	850914	A	C	382	1500 bp unstream region	Hypothetical protein
POX04280		NA	NA	NA	NA	scaffold_3	2616147	C	T	1490	1500 bp unstream region	Hypothetical protein
POX05515		NA	NA	NA	NA	scaffold_4	2861314	A	G	9	1500 bp unstream region	Hypothetical protein
POX05833		NA	NA	NA	NA	scaffold_5	523619	A	C	567	1500 bp unstream region	Hypothetical protein
POX05962		NA	NA	NA	NA	scaffold_5	935733	G	C	1208	1500 bp unstream region	Hypothetical protein
POX06075		NA	NA	NA	NA	scaffold_5	1319822	G	A	510	1500 bp unstream region	Hypothetical protein
POX06167		NA	NA	NA	NA	scaffold_5	1608695	C	T	25	1500 bp unstream region	Hypothetical protein
POX06624		NA	NA	NA	NA	scaffold_6	50988	A	G	359	1500 bp unstream region	Hypothetical protein
POX06672		NA	NA	NA	NA	scaffold_6	297934	G	A	36	1500 bp unstream region	Hypothetical protein
POX06673		NA	NA	NA	NA	scaffold_6	299128	G	A	725	1500 bp unstream region	Hypothetical protein
POX06952		NA	NA	NA	NA	scaffold_6	1154831	T	C	133	1500 bp unstream region	Hypothetical protein

POX06983		NA	NA	NA	NA	scaffold_6	1240576	G	A	72	1500 bp unstream region	Endo-1,4-β-glucanase
POX07319		NA	NA	NA	NA	scaffold_6	2275900	C	T	945	1500 bp unstream region	Hypothetical protein
POX07574		NA	NA	NA	NA	scaffold_7	558780	G	A	188	1500 bp unstream region	Hypothetical protein
POX07746		NA	NA	NA	NA	scaffold_7	1171285	G	A	333	1500 bp unstream region	Hypothetical protein
POX07920		NA	NA	NA	NA	scaffold_7	1761971	A	G	172	1500 bp unstream region	Hypothetical protein
POX07962		NA	NA	NA	NA	scaffold_8	128706	G	A	956	1500 bp unstream region	Hypothetical protein
POX08241		NA	NA	NA	NA	scaffold_8	912157	T	C	656	1500 bp unstream region	Hypothetical protein
POX09581		NA	NA	NA	NA	scaffold_15	129370	C	T	184	1500 bp unstream region	Aminoglycoside phosphotransferase
POX09581		NA	NA	NA	NA	scaffold_15	129610	G	A	424	1500 bp unstream region	Aminoglycoside phosphotransferase
POX09581	129178	C<->T	GAC<->GAT	D<->D	Synonymous	scaffold_15	129370	C	T	184	1500 bp unstream region	Aminoglycoside phosphotransferase
POX09581	NA	NA	NA	NA	Synonymous	scaffold_15	129610	G	A	424	1500 bp unstream region	Aminoglycoside phosphotransferase
POX09581	NA	NA	NA	NA	Synonymous	scaffold_15	129633	A	G	447	1500 bp unstream region	Aminoglycoside phosphotransferase
POX09616		NA	NA	NA	NA	scaffold_15	236294	C	G	61	1500 bp unstream region	Hypothetical protein
POX09626		NA	NA	NA	NA	scaffold_15	263643	A	G	1058	1500 bp unstream region	DNA breaking-rejoining enzyme, catalytic core
POX09797		NA	NA	NA	NA	scaffold_19	65416	C	T	142	1500 bp unstream region	Hypothetical protein
POX09813		NA	NA	NA	NA	scaffold_25	3072	T	C	296	1500 bp unstream region	Hypothetical protein
POX09820		NA	NA	NA	NA	scaffold_28	6907	A	G	536	1500 bp unstream region	Hypothetical protein
POX00799		NA	NA	NA	NA	scaffold_1	2405438	T	G	53	300 bp downstream region	Hypothetical protein
POX02617		NA	NA	NA	NA	scaffold_2	2119426	C	T	28	300 bp downstream region	Hypothetical protein
POX03348		NA	NA	NA	NA	scaffold_2	4341229	G	A	256	300 bp downstream region	Hypothetical protein
POX05545		NA	NA	NA	NA	scaffold_4	2964519	T	C	98	300 bp downstream region	Hypothetical protein
POX05546		NA	NA	NA	NA	scaffold_4	2964774	G	A	186	300 bp downstream region	Hypothetical protein
POX06888		NA	NA	NA	NA	scaffold_6	961348	T	C	89	300 bp downstream region	Hypothetical protein
POX06955		NA	NA	NA	NA	scaffold_6	1161505	C	T	31	300 bp downstream region	Hypothetical protein
POX06991		NA	NA	NA	NA	scaffold_6	1262510	G	A	93	300 bp downstream region	Hypothetical protein
POX08720		NA	NA	NA	NA	scaffold_10	60890	G	A	105	300 bp downstream region	Hypothetical protein
POX09799		NA	NA	NA	NA	scaffold_20	8813	A	G	13	300 bp downstream region	Hypothetical protein
POX00082	257332	A<->C	ATC<->CTC	I<->L	Nonsynonymous	NA	NA	NA	NA	NA		Hypothetical protein
POX00715	2174681	G<->A	GTG<->ATG	V<->M	Nonsynonymous	NA	NA	NA	NA	NA		Hypothetical protein
POX01113	3467516	A<->C	ACC<->CCC	T<->P	Nonsynonymous	NA	NA	NA	NA	NA		Ribosomal protein S15
POX01133	3535181	G<->C	AAG<->AAC	K<->N	Nonsynonymous	NA	NA	NA	NA	NA		Ribosomal protein S13
POX01395	4313388	G<->A	CCG<->CCA	P<->P	Synonymous	NA	NA	NA	NA	NA		Hypothetical protein
POX01661	5072141	G<->A	GAT<->AAT	D<->N	Nonsynonymous	NA	NA	NA	NA	NA		Hypothetical protein
POX01868	5682577	C<->T	ACT<->ATT	T<->I	Nonsynonymous	NA	NA	NA	NA	NA		Hypothetical protein
POX02118	598859	G<->A	GAA<->AAA	E<->K	Nonsynonymous	NA	NA	NA	NA	NA		Hypothetical protein
POX02669	2276059	A<->C	TCA<->TCC	S<->S	Synonymous	NA	NA	NA	NA	NA		Hypothetical protein
POX02883	2932538	G<->A	GAA<->AAA	E<->K	Nonsynonymous	NA	NA	NA	NA	NA		Zn2Cys6 transcription factor
POX02958	3163067	G<->A	GTA<->ATA	V<->I	Nonsynonymous	NA	NA	NA	NA	NA		Calcium/calmodulin-dependent protein kinase
POX03359	4368973	T<->C	TAG<->CAG	*<->Q	Stop_nonsyn	NA	NA	NA	NA	NA		Hypothetical protein
POX03494	106520	G<->A	CGA<->CAA	R<->Q	Nonsynonymous	NA	NA	NA	NA	NA		Hypothetical protein
POX03641	537799	C<->T	CTC<->TTC	L<->F	Nonsynonymous	NA	NA	NA	NA	NA		Beta-glucosidase
POX04052	1920278	G<->A	TGC<->TAC	C<->Y	Nonsynonymous	NA	NA	NA	NA	NA		Hypothetical protein
POX04165	2273645	C<->T	GAC<->GAT	D<->D	Synonymous	NA	NA	NA	NA	NA		Hypothetical protein
POX04725	575782	G<->A	GAG<->AAG	E<->K	Nonsynonymous	NA	NA	NA	NA	NA		HLH transcription factor
POX04922	1161213	C<->T	AAC<->AAT	N<->N	Synonymous	NA	NA	NA	NA	NA		Hypothetical protein
POX05027	1435067	C<->T	CCC<->TCC	P<->S	Nonsynonymous	NA	NA	NA	NA	NA		Hypothetical protein
POX05164	1846551	C<->T	TCA<->TTA	S<->L	Nonsynonymous	NA	NA	NA	NA	NA		Hypothetical protein
POX05375	2463508	G<->A	CTG<->CTA	L<->L	Synonymous	NA	NA	NA	NA	NA		Hypothetical protein
POX05609	3138853	G<->C	GTC<->CTC	V<->L	Nonsynonymous	NA	NA	NA	NA	NA		Hypothetical protein
POX05751	269546	G<->A	GTT<->ATT	V<->I	Nonsynonymous	NA	NA	NA	NA	NA		Hypothetical protein
POX05990	1020432	C<->T	TCG<->TTG	S<->L	Nonsynonymous	NA	NA	NA	NA	NA		Permease
POX06859	864182	C<->T	CCC<->CCT	P<->P	Synonymous	NA	NA	NA	NA	NA		Putative glycogenin
POX07343	2350315	G<->A	ATG<->ATA	M<->I	Nonsynonymous	NA	NA	NA	NA	NA		Hypothetical protein
POX07776	1269957	C<->T	GGC<->GGT	G<->G	Synonymous	NA	NA	NA	NA	NA		Hypothetical protein
POX08425	1464880	G<->A	GAG<->GAA	E<->E	Synonymous	NA	NA	NA	NA	NA		Hypothetical protein
POX08600	338121	G<->A	GAG<->AAG	E<->K	Nonsynonymous	NA	NA	NA	NA	NA		Hypothetical protein
POX09009	354387	G<->A	GAG<->AAG	E<->K	Nonsynonymous	NA	NA	NA	NA	NA		Hypothetical protein

POX09060	487543	C<->T	ACA<->ATA	T<->I	Nonsynonymous	NA	NA	NA	NA	NA		Hypothetical protein
POX09438	485864	C<->T	CGC<->TGC	R<->C	Nonsynonymous	NA	NA	NA	NA	NA		Hypothetical protein
POX09549	12109	C<->A	GCA<->GAA	A<->E	Nonsynonymous	NA	NA	NA	NA	NA		Hypothetical protein
POX09802	45404	T<->G	GGT<->GGG	G<->G	Synonymous	NA	NA	NA	NA	NA		Pectin lyase
