# Supplementary material for: ARTP/EMS-combined multiple mutagenesis efficiently improved production of raw starch-degrading enzymes in Penicillium oxalicum and characterization of the enzyme-hyperproducing mutant
Source: Biotechnol Biofuels. 2020 Nov 11;13:187. doi: 10.1186/s13068-020-01826-5 (PMC7661180; doi:10.1186/s13068-020-01826-5)
Supplement: Supplementary file 6 — Additional file 6: Table S2. Mutation sites in coding sequences (CDS) in the mutant A2-13 compared with that in OXPoxGA15A. [file 13068_2020_1826_MOESM6_ESM.pdf]

Additional file 6: Table S2. Mutation sites in coding sequences (CDS) in the mutant A2-13 compared with that in the OX*PoxGA15A*

Gene	Locus	Mutation	AA change	Products
POX00063	197194	Deletion G	Shift	endo-β-1,4-xylanase
POX02669	2276066	Deletion CTCCCG	HSR <->H	alkaline phosphatase
POX07393	877	Deletion G	ALLP <->A	ABC transporter
	878	Deletion TACTCCCC		
POX09551	17671	Insertion A	Shift	hypothetical protein
	17697	Insertion G		
