# Supplementary material for: ARTP/EMS-combined multiple mutagenesis efficiently improved production of raw starch-degrading enzymes in Penicillium oxalicum and characterization of the enzyme-hyperproducing mutant
Source: Biotechnol Biofuels. 2020 Nov 11;13:187. doi: 10.1186/s13068-020-01826-5 (PMC7661180; doi:10.1186/s13068-020-01826-5)
Supplement: Supplementary file 7 — Additional file 7: Table S3. Primers used in this study. [file 13068_2020_1826_MOESM7_ESM.pdf]

Additional file 7: Table S3 Primers used throughout this study

**Name**	**Sequence (5’-3’)**
PoxGA15A-F	CCTCGGTGAGCCCAAGTT
PoxGA15A-R	CCAAAGTCAATCAAGGCAA
POX02412-F	TATGTGGATTCCTTCCGCTCTA
POX02412-R	ATGGATTGCCTCCTTGGT
PoxAmy13A-F	CTGACGGCTGCCCAATG
PoxAmy13A-R	CCAAATCGCAGTAAATCCC
PoxAmyR-F	ACCCAGCCAGGGAACCAC
PoxAmyR-R	CATTCCGATGCCGTGAGC
Actin-F	CTCCATCCAGGCCGTTCTG
Actin-R	CATGAGGTAGTCGGTCAAGTCAC
